# Common Bean Suppresses Hepatic Ceramide Metabolism in a Mouse Model of Metabolic Dysfunction-Associated Steatotic Liver Disease

**DOI:** 10.3390/nu16183196

**Published:** 2024-09-21

**Authors:** Vanessa K. Fitzgerald, Tymofiy Lutsiv, John N. McGinley, Elizabeth S. Neil, Mary C. Playdon, Henry J. Thompson

**Affiliations:** 1 Cancer Prevention Laboratory, Colorado State University, Fort Collins, CO 80523, USA; vanessa.fitzgerald@colostate.edu (V.K.F.); tymofiy.lutsiv@colostate.edu (T.L.);; 2 Department of Nutrition and Integrative Physiology, University of Utah, Salt Lake City, UT 84112, USA; mary.playdon@hci.utah.edu

**Keywords:** ceramides, common bean, lipid metabolism, metabolic dysfunction-associated steatotic liver disease, metabolomics, serine palmitoyl transferase, sphingolipids

## Abstract

**Background/Objectives:** The incidence of metabolic dysfunction-associated steatotic liver disease (MASLD), a condition linked to the ongoing obesity pandemic, is rapidly increasing worldwide. In turn, its multifactorial etiology is consistently associated with low dietary quality. Changing dietary macronutrient and phytochemical quality via incorporating cooked common bean into an obesogenic diet formulation has measurable health benefits on the occurrence of both obesity and hepatic steatosis in C57BL/6 mice. **Methods:** A cohort of C57BL/6 mice were randomized into experimental diets containing multiple dietary concentrations of common bean. The primary endpoint of this study was comparing metabolomic analyses from liver and plasma of different treatment groups. Additionally, RNA sequencing and protein expression analysis via nanocapillary immunoelectrophoresis were used to elucidate signaling mediators involved. **Results:** Herein, global metabolomic profiling of liver and plasma identified sphingolipids as a lipid subcategory on which bean consumption exerted significant effects. Of note, C16 and C18 ceramides were significantly decreased in bean-fed animals. Hepatic RNAseq data revealed patterns of transcript expression of genes involved in sphingolipid metabolism that were consistent with metabolite profiles. **Conclusions:** Bean incorporation into an otherwise obesogenic diet induces effects on synthesis, biotransformation, and degradation of sphingolipids that inhibit the accumulation of ceramide species that exert pathological activity. These effects are consistent with a mechanistic role for altered sphingolipid metabolism in explaining how bean inhibits the development of MASLD.

## 1. Introduction

Metabolic dysfunction-associated steatotic liver disease (MASLD) is considered an intermediary metabolic phenotype in the continuum between excess accumulation of lipids in adipose depots and the occurrence of insulin resistance, type 2 diabetes, metabolic syndrome, cardiovascular disease, and hepatocellular carcinoma [1,2,3]. Bibliometric analyses and systematic reviews of the relevant peer-reviewed literature suggest a dynamic interplay in the development of these morphometric and metabolic phenotypes that is dependent in part on the magnitude of lipid accumulation in various fat depots and in the liver [4,5].

MASLD, the prevalence of which is rapidly increasing globally [6], progresses to metabolic dysfunction-associated steatohepatitis (MASH). MASH is distinguished from MASLD by inflammation that can lead to cell damage, fibrosis, and progressive liver damage that can eventually cause cirrhosis as well as hepatocellular carcinoma [7]. Though it was only recently that the FDA approved the first drug for the treatment of MASH, lifestyle management remains a component of the first-line treatment of MASH, particularly high-quality diet and exercise [8,9,10,11]. 

Relative to a high-quality diet, dietary fiber consumption shows inverse relationships with the development of MASLD [12,13]. An under-appreciated possibility emerging from population-based studies of food consumption patterns is that consuming common beans and other pulses, i.e., grain legumes that are rich in dietary fiber, can protect against a range of chronic diseases associated with the development and progression of MASLD [14,15,16,17]. However, a limitation of these data sources is the lack of specificity of most food frequency questionnaires in discriminating between specific pulses versus overall legume consumption. This situation obscures the contributions of various pulses and the dietary level of intake (daily dose) required to obtain health benefits. These limitations are key considerations in interpreting research on pulse crops, including the common bean. 

To overcome some of these challenges, we have leveraged preclinical models to deconstruct potentially important epidemiological findings to inform understanding of effective dose, the chemical mediators, and the molecular mechanisms that account for health benefits. The data-driven investigation reported herein focused on using global metabolomic profiling to identify candidate chemical mediators associated with protection against MASLD by bean consumption and to infer potential regulators of observed metabolite changes using RNAseq analysis of the liver. 

## 2. Materials and Methods

### 2.1. Animal Feeding Study

The animal protocol has been previously reported [18,19]. Briefly, twenty-day-old male and female C57BL6/J mice (cat#000664) were received from the Jackson Laboratory (Barr Harbor, ME, USA). Animals were maintained via standard husbandry conditions. Mice were housed in solid-bottom polycarbonate mouse cages and had ad libitum access to filtered water and food. They were exposed to a 12 h light/dark cycle and 27.5 ± 2 °C ambient temperature. Animals were adapted to the husbandry routine and a purified powdered diet (based on formulation D12266B by Research Diets Inc., New Brunswick, NJ, USA) containing 32.5% kcal fat, as described in [20], until eight weeks of age, at which time 20 animals of each biological sex were assigned to their respective experimental diet groups by staggered randomization by body weight and fed their respective diet for 12–14 weeks. At the end of the study, animals were anesthetized via isoflurane inhalation to the surgical plane, and blood was obtained while animals were under anesthesia via cardiac puncture using a 25 or 26 g needle and 1 cc syringe. The blood was transferred into an EDTA-coated microtainer tube spun at 2000× *g* for 10 min, after which the plasma was isolated and transferred to a cryovial and snap-frozen in liquid nitrogen. The liver was excised, placed in a sample bag, and snap-frozen in liquid nitrogen. The liver and plasma samples were stored at −70 °C until subsequent evaluation. All animal work was approved by the CSU IACUC (Colorado State University, Institutional Animal Care and Use Committee protocol number 1431).

### 2.2. Experimental Approach

The overall experimental study design has been previously reported [18] and, for convenience, is shown in Supplemental Table S1. Briefly, both male and female mice were studied at multiple dietary concentrations of the common bean. The central focus of this study was on metabolomic analyses of the liver and plasma. Given that the prevalence of MASLD is higher in males than females [21,22], the public health impact may be greatest in men, who also suffer from a larger dietary fiber gap than females [23]. Therefore, our initial focus was on male animals. To that end, 7 pooled male mouse samples (each pool contained equal amounts of sample from two mice) per diet group were evaluated, a number recommended by Metabolon, Inc. (Morrisville, NC, USA), based on their experience in preclinical models. We consider this a reasonable approach given the difficulty of using power analysis to determine the sample size for metabolomic studies, as detailed in [24], and because of the per-sample cost of this analysis. To gain insight into the metabolomic response in female mice at the same dietary concentration of the common bean, pools of liver and plasma from 14 mice were created and evaluated. RNAseq analysis was performed in both males and females at three dietary concentrations of bean using 10 pools (two mice per pool) per dietary group for each biological sex.

### 2.3. Diet Composition

Experimental diets were formulated to remain equal in total dietary energy content, thus enabling evaluation of the direct effects of the bean. Equal macronutrient proportions were used to compose all diets, weight by weight, i.e., 58.6% total carbohydrate, 20% total protein, 15.5% total fat, and 5.9% micronutrients, according to the AIN-93G recommendations. Mice received 32.5% kcal derived from milk fat, which is within the range for normal lipid dietary practice in humans [23] and obesogenic studies using the C57BL6/J animal model [25]. All diets were comprised of purified ingredients except for the bean. The white kidney beans were cooked as a whole food, processed with leachate, puréed, freeze-dried, and then milled into a homogenous fine powder. The total dietary protein derived from the bean was calculated as 0%, 17.5%, 35%, and 70%. The total dietary fiber concentration of the bean was 23.5 g/100 g bean powder [22]. The work herein reports the results from 0% (bean-free control) and 70% (labeled as just “bean”) groups only (see Section 3). The formulations of these experimental diets have been previously reported [18], but for convenience, they are in Supplemental Table S2.

### 2.4. Metabolomics Analysis

EDTA plasma (150 µL) and liver (100 mg) were collected from 28 male animals total, 14 fed bean-free control and 14 fed 70% protein from beans. To maximize statistical power while remaining cost-effective, samples from 2 animals of the same treatment were pooled, resulting in male *n* = 7 per treatment. Males were the primary focus of the study; however, samples from 14 female animals fed the same treatment were pooled for both plasma and liver (female *n* = 1 per treatment) to give insight into sex differences of metabolite profiles. Samples were delivered to Metabolon, Inc. (Morrisville, NC, USA), for metabolomic analysis via ultrahigh-performance liquid chromatography-tandem mass spectroscopy (UPLC-MS/MS). Metabolon used a Waters ACQUITY ultra-performance liquid chromatography (UPLC) with a Thermo Scientific (Waltham, MA, USA) Q-Exactive high resolution/accurate mass spectrometer interfaced with a heated electrospray ionization (HESI-II) source and Orbitrap mass analyzer operated at 35,000 mass resolution [26]. Samples were analyzed using Metabolon’s global untargeted metabolomics platform (HD4). Before HD4 platform analysis, an equivalent ratio of mass-to-extraction buffer was used for liver tissue samples. For plasma samples, the values for each biochemical were normalized based on the volume of the sample extracted. The resulting data were median-scaled, and missing values were imputed with sample set minimums, each on a per biochemical basis. 

### 2.5. RNA Isolation and RNAseq Analysis

These procedures have been explained in detail previously [18]. Briefly, frozen liver (*n* = 10 per treatment) was ground to powder using ceramic mortar and pestles cooled with liquid nitrogen. RNA was isolated following the manufacturer’s protocol using an RNeasy mini-kit cat. 74,104 (QIAGEN, Inc., Germantown, MD, USA). RNA integrity was determined using an Experion Automated Electrophoresis Station (Bio-Rad Laboratories, Inc., Hercules, CA, USA). The RNA samples were submitted for cDNA library construction and RNA sequencing (Illumina, Inc., San Diego, CA, USA) to the Genomics and Microarray Core at the University of Colorado-Anschutz medical campus (Aurora, CO, USA). Raw sequencing data were processed using CLC Genomics Workbench software, version 21.0.3 (QIAGEN, Redwood City, CA, USA), and the resulting differentially expressed genes (DEGs) were uploaded to Ingenuity Pathway Analysis (IPA) software, v81348237, for subsequent analysis (QIAGEN, Redwood City, CA, USA). There were 3688 Core Analysis-ready genes that were differentially expressed between the bean and control filtered by a *p*-value < 0.05 and the maximum mean group expression intensity >1.0. The Core Analysis engine was utilized to interpret expression patterns of DEGs data. Activation *z*-scores, used to predict patterns of expressed genes between groups, were deemed significant with a *z*-score ≥ 2 for activation and ≤−2 for inhibition.

### 2.6. Western Blot-Based Nanocapillary Electrophoresis

The liver was ground under liquid nitrogen to a powder and weighed into pre-cooled microfuge tubes using the same tissue pooling strategy listed in Section 2.4. Protein lysates of the pooled tissue samples were prepared as previously described [20]. Protein lysate concentration was determined by the BCA method [27]. Nanocapillary immunoelectrophoresis was performed using a Jess instrument (ProteinSimple, San Jose, CA, USA) as previously described [28] with the following changes: the final concentration of the protein lysates was 0.7 mg/mL, and each primary antibody was incubated for 120 min. The following primary antibodies were used: rabbit anti-LASS6/CERS6 (cat. PA5-20648), rabbit anti-SPTLC2 (cat. PA5-31130) from Thermo Fisher Scientific (Waltham, MA, USA), and rabbit anti-DEGS1 (cat. SAB2100559) from MilliporeSigma (St. Louis, MO, USA). Data were normalized by dividing target protein peak area by the corrected total protein area of the sample within each capillary.

### 2.7. Statistical Analysis

For metabolomics data, peak areas were median-scaled and missing values were imputed with the minimum observed value of each compound. Plasma data were additionally normalized to the extracted volume. Scaled, imputed, and normalized data were transformed using a natural log to perform statistical testing. Specifically, Welch’s two-sample *t*-test was used to identify significantly different biochemicals between experimental groups, with *p*-values < 0.05 and False Discovery Rate (FDR) *q*-values < 0.1 considered statistically significant [29]. Principal coordinates analysis (PCoA) was utilized to explore global trends in the dataset. Non-transformed normalized peak areas were used to calculate means and respective fold change values between experimental groups. Statistical evaluation of RNAseq data in CLC Genomics Workbench and IPA has been previously reported [18]. A selection of DEGs and metabolites within ceramide metabolic pathways were further analyzed separately via PCoA. Additional visualizations were performed in R, version 4.4.0, using ggpubr, rstatix, and tidyverse packages.

## 3. Results

**Figure 1:**
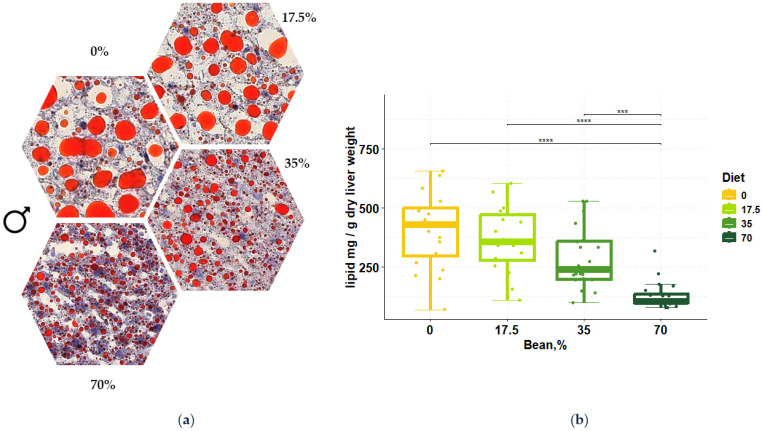
Bean prevents the accumulation of lipid droplets in the liver of male animals in a dose-dependent manner. (**a**) Oil Red O- and hematoxylin-stained liver sections across different diets: percentage indicates the amount of total dietary protein derived from common bean. Extracellular and intracellular lipid is stained red and hepatic nuclei are stained blue. (**b**) Box plots of the bean dose effect on the hepatic lipid. Values represent the amount of lipid in mg normalized to g of dry liver weight across the diet groups. Groups indicate total dietary protein percent sourced by beans. Kruskal–Wallis testing showed significant differences in the diet effect (χ^2^ = 36.09, *p*-value = 7.167 × 10^−8^) with the large effect size (*η*^2^ = 0.435). Pairwise comparisons between the diet groups were conducted using the post-hoc Dunn test: *** *q*-value < 0.001; **** *q*-value < 0.0001 (adapted from [18]).

Previously, we have reported that consumption of beans at amounts ranging from 35% to 70% of total dietary protein was effective in reducing levels of fat in the liver of male and female C57BL/6 mice compared with animals fed an equivalent isocaloric bean-free control (Figure 1 and Supplementary Figure S1 [18]). A significant reduction trend was observed even at the 35% dose, albeit not reaching the statistical cutoff after the multiple test correction (*z* = −2.19, *p*-value = 0.028, and *q*-value = 0.056). Thus, to deepen our understanding of bean effects in the liver of male mice, our focus was on a 0% and 70% bean contrast, labeled as “control” and “bean” henceforth. Supplementary Materials contain additional information from other doses of beans and from female mice. 

### 3.1. Metabolomic Profiles Reveal Sphingolipid Metabolism Affected

**Figure 2:**
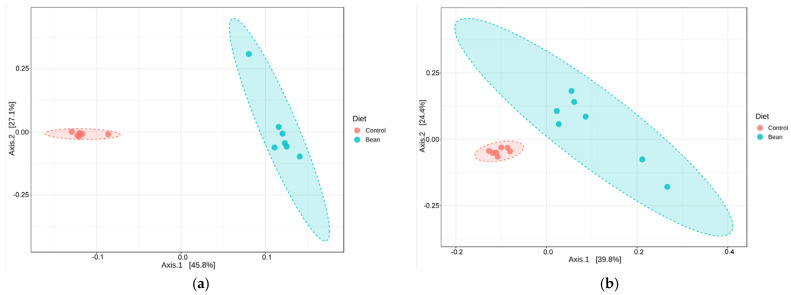
PCoAs of metabolomic profiles in liver (**a**) and plasma (**b**). Ordination was performed with the Bray–Curtis dissimilarity index, and 95% confidence intervals are shown as ovals.

The plasma and liver tissue metabolite datasets comprise a total of 1024 metabolites (919 named and 105 unnamed metabolites) and 1065 metabolites (958 named and 107 unnamed metabolites), respectively. Principal coordinates analysis (PCoA) can be used to quickly assess the magnitude of the effect of experimental conditions compared to each other and population variability. Samples with similar biochemical profiles cluster together, whereas samples with distinct profiles segregate away from one another. In this study, the diet composition (0% and 70% bean protein) appears to drive clear separation between the study groups (Figure 2) in both matrices (liver and plasma), pointing to distinct metabolomic profiles. PERMANOVA analysis confirmed the separation between the groups in the liver (*R*^2^ = 0.439, pseudo-*F*-value = 9.376, and *p*-value = 0.003) and plasma (*R*^2^ = 0.336, pseudo-*F*-value = 6.074, and *p*-value = 0.003). 

A summary of the number of metabolites that achieved nominal statistical significance (*p* < 0.05) is shown below (Table 1); *q* < 0.1 signifies statistically significant findings while accounting for multiple metabolites. Using steatosis as the phenotype of the study, metabolites within the lipid super pathway became a natural focus for differential response to bean treatment. Lipids account for ~50% of differentially observed metabolites overall (49.28% in liver, 50.75% in plasma). We observed a larger proportion of decreased lipids in the bean group (60.36% in liver, 57.8% in plasma; Table 1). 

### 3.2. De Novo Ceramide Synthesis

**Figure 3:**
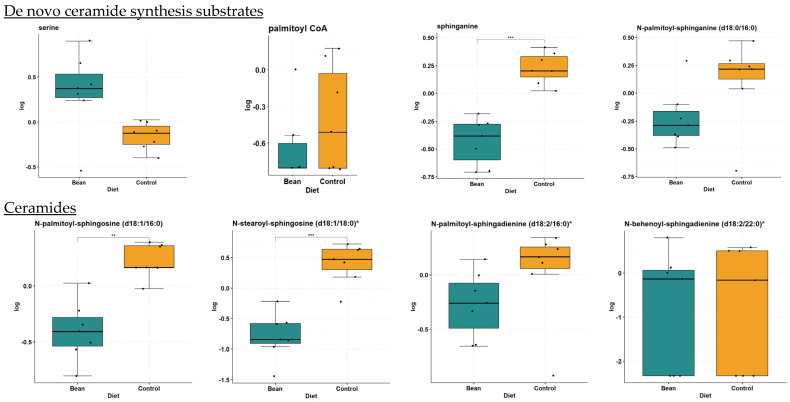

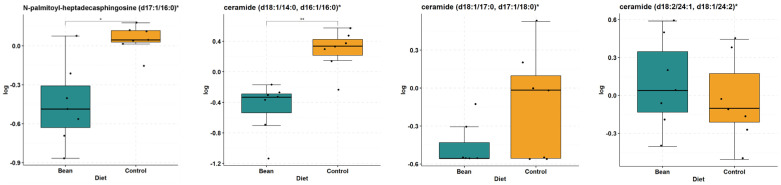
Box plots of metabolites involved in de novo ceramide synthesis and resulting ceramides in liver. Differences indicated where * *q*-value < 0.05; ** *q*-value < 0.01; *** *q*-value < 0.001.

In liver samples, there is significant reduction of many ceramides in the bean group with the largest differences occurring in *N*-stearoyl-sphingosine (d18:1/18:0) (FC = 0.31, *p*-value = 4.82 × 10^−5^, and *q*-value = 4.8 × 10^−4^) and ceramide (d18:1/14:0, d16:1/16:0) (FC = 0.48, *p*-value = 7.03 × 10^−4^, and *q*-value = 3.8 × 10^−3^). The de novo synthesis pathway is suppressed in bean-fed animals, as shown by the reduction of ceramides and directly upstream precursors found in the liver (Table 2, Figure 3). This reduction is not due to limited substrates at the beginning of ceramide de novo synthesis as serine and palmitoyl-CoA are increased or unchanged, respectively, in bean-fed animals (FC = 1.74, *p*-value = 0.028, and *q*-value = 0.067 and FC = 0.773, *p*-value = 0.314, and *q*-value = 0.415). Progression through de novo synthesis shows that sphinganine (FC = 0.52, *p*-value = 3.24 × 10^−5^, and *q*-value = 3.79 × 10^−4^) and dihydroceramide (*N*-palmitoyl-sphinganine (d18:0/16:0)) (FC = 0.7, *p*-value = 0.08, and *q*-value = 0.156) are reduced as well as ceramides containing 16:0, 18:0, 20:0, 22:0, and 24:1/2 acyl groups in liver metabolite profiles. Liver ceramides with acyl groups 24:1 and 24:2 did not show any difference between treatment groups in the liver but showed reduced amounts in the plasma (see Section 3.6).

### 3.3. Salvage Pathway of Ceramide Biotransformation and Degradation

**Figure 4:**
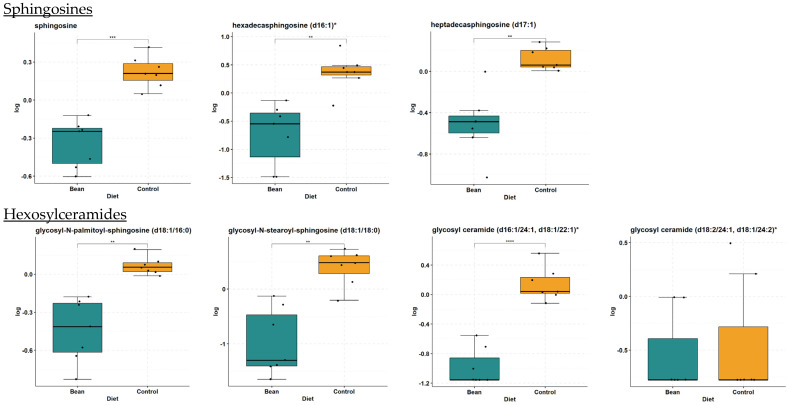
Box plots of sphingosine and HCER metabolites involved in salvage pathway in liver. Differences indicated where * *q*-value < 0.05; ** *q*-value < 0.01; *** *q*-value < 0.001; **** *q*-value < 0.0001.

Our data show significant decreases in sphingosines (FC = 0.57, *p*-value = 4.17 × 10^−5^, and *q*-value = 4.4 × 10^−4^) and hexosylceramides (HCER; i.e., glycosyl-*N*-stearoyl-sphingosine (d18:1/18:0): FC = 0.29, *p*-value = 4.8 × 10^−4^, and *q*-value = 2.9 × 10^−3^) in the liver tissue of bean-fed animals. All detectable sphingosines and HCER are shown in Table 3 with associated box plots (Figure 4). These data implicate bean treatment in modulating the salvage pathway of ceramide regeneration. 

### 3.4. Sphingomyelin Pathway of Ceramide Biotransformation

**Figure 5:**
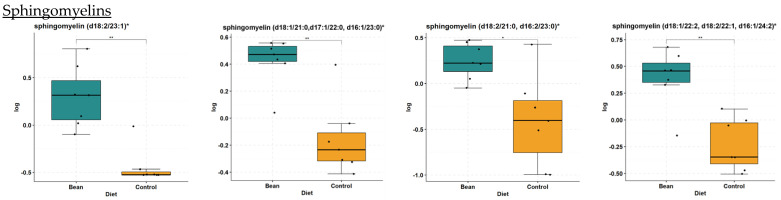
Box plots of selected sphingomyelin metabolites from the sphingomyelin hydrolysis pathway in liver. Differences indicated where * *q*-value < 0.05; ** *q*-value < 0.01.

Sphingomyelins are increased in the bean-fed group (Table 4). Out of 26 sphingomyelins and dihydrosphingomyelins detected in the liver samples, 9 increased in the bean treatment (i.e., sphingomyelin (d18:2/23:1) FC = 2.16, *p*-value = 4.64 × 10^−4^, and *q*-value = 2.84 × 10^−3^). Selected box plots are shown in Figure 5. The other 17 metabolites were not nominally significantly different between treatments. These data suggest that bean treatment does not activate the sphingomyelin hydrolysis pathway leading to ceramide regeneration but rather indicates ceramide biotransformation into endpoint sphingomyelins. 

### 3.5. Transcriptional Regulation Altered by Bean Consumption 

**Figure 6:**
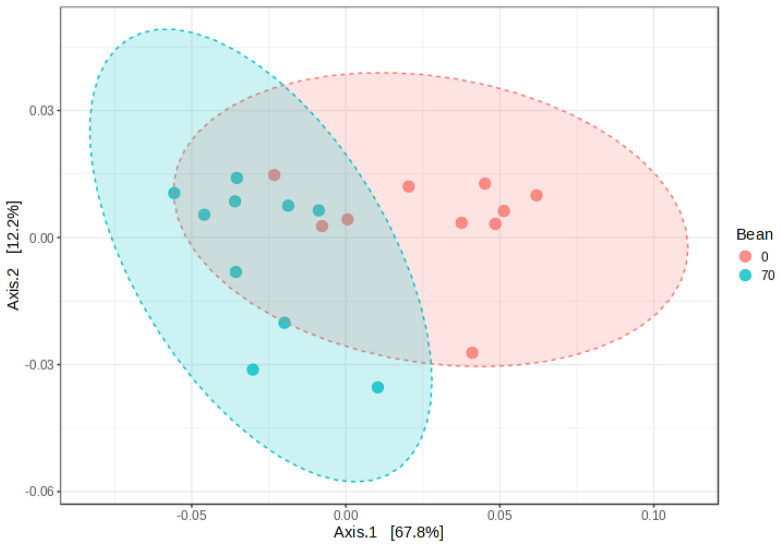
PCoA of the selection of genes within the Ceramide Biosynthesis canonical pathway from IPA (de novo ceramide synthesis). Ordination was performed with the Bray–Curtis dissimilarity index, and 95% confidence intervals are shown as ovals. PERMANOVA testing indicated *R*^2^ = 0.42, pseudo-*F*-value =12.876, and *p*-value = 0.001.

We complemented the metabolomic analysis with the evaluation of RNAseq data to assess anabolic/catabolic pathways underlying observed changes in sphingolipid metabolite profiles. RNAseq data from the liver tissue samples were analyzed using Ingenuity Pathway Analysis (IPA) software, which utilized the curated extensive QIAGEN Knowledge Base to interpret gene expression patterns functionally. Consumption of beans revealed patterns of differentially expressed genes (DEGs) involved in regulating sphingolipid metabolism. When transcriptomic data from all involved genes were subjected to principal coordinates analysis (PCoA) of dissimilarity-based Bray–Curtis distances, clear separation was observed between control and bean groups in (Figure 6). PERMANOVA analysis confirmed the distinction between the groups with *R*^2^ = 0.42, pseudo-*F*-value =12.876, and *p*-value = 0.001.

**Figure 7:**
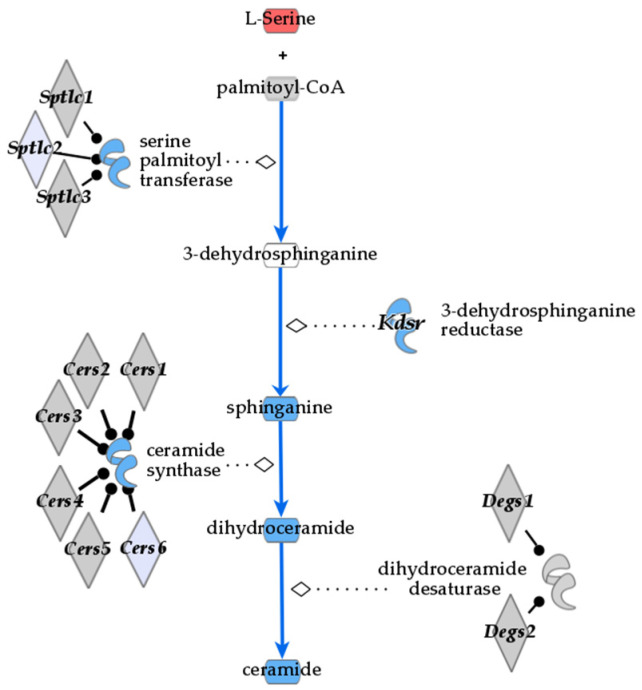
De novo ceramide biosynthesis pathway with an overlay of transcriptomic and metabolomic data. Blue indicates observed reduction, red—increase, gray—statistically insignificant result, and white—lack of observation in bean compared to control.

DEG patterns indicate suppression of de novo biosynthesis of ceramides in the liver of bean-fed mice. Serine C-palmitoyl transferase (Spt) uses activated fatty acid palmitoyl-CoA to convert L-serine into 3-dehydrosphinganine, and the latter is further metabolized by 3-dehydrosphinganine reductase (Kdsr) into NADP+ and sphinganine (Table 5 and Figure 7). *Sptlc2* and *Kdsr* were downregulated by beans, supporting metabolomics findings of lower sphinganine (FC = 0.52, *p*-value = 3.24 × 10^−5^, and *q*-value = 3.79 × 10^−4^), yet increased levels of serine (FC = 1.74, *p*-value = 0.028, and *q*-value = 0.067). *Cers6* was the only ceramide synthase downregulated by bean consumption. Dihydroceramide desaturases (*Degs1* and *Degs2*) were not significantly different in expression between treatments. Because Sptlc2, Cer6, and Degs1 are widely studied regulators of sphingolipid metabolism, their protein data (transcripts and protein) were examined but failed to provide convincing evidence that this type of “snapshot” assessment has the sensitivity to detect how bean consumption is impacting sphingolipid metabolism (Supplementary Figures S5 and S6).

**Figure 8:**
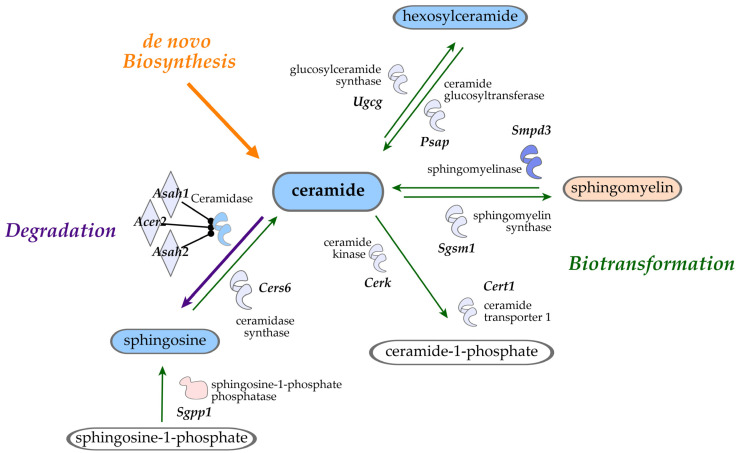
Overlook of ceramide metabolism and bean-induced effects. Ceramide metabolism includes degradation into sphingosine and biotransformation into cermide-1-phosphate, sphingomyelin, and hexosylceramide. Metabolon metabolites and RNAseq gene transcription data are overlaid: blue shows decrease while red shows increase in bean compared with the control.

Moreover, ceramide transporter (*Cert1*) and kinase (*Cerk*) were also expressed less in the bean group, predicting inhibition of ceramide 1-phosphate production (Table 6). Ceramide levels also could not be regenerated in the salvage pathway either from glucosylceramides (HCER) as glucosidase (*Psap*) and UDP-glucose-ceramide glucosyltransferases (*Ugcg*), or from sphingosine-1-phosphate as the phosphatase of the latter was upregulated (*Sgpp1*) and levels of sphingosine were downregulated (FC = 0.57, *p*-value = 4.17 × 10^−5^, and *q*-value = 4.4 × 10^−4^) in bean versus control. Ceramidases (*Asah1*, *Asah2*, and *Acer2*) were also decreased in the bean group, further supporting that ceramides were most likely not degraded to produce sphingosine molecules. Interestingly, expression levels of sphingosine-1-phosphate (S1P) receptors 1 and 5 (*S1pr1:* log_2_FC = 0.61, *p*-value = 1.26 × 10^−12^, and *q*-value = 1.78 × 10^−10^; *S1pr5:* log_2_FC = 0.89, *p*-value = 2 × 10^−6^, and *q*-value = 6.14 × 10^−5^) were slightly increased in the bean group, while levels of *S1pr2* were significantly reduced compared with control (log_2_FC = −0.71, *p*-value = 2.69 × 10^−8^, and *q*-value = 1.45 × 10^−6^). *S1pr2* binds sphingosine-1-phosphate (S1P) and has a downstream effect on PI3K-AKT signaling, predicted to be downregulated in IPA (activation *z*-score = −2.611 and *p*-value = 4.21 × 10^−3^). Despite such discrepancy, downstream DEG patterns strongly predicted *S1rp2* and *S1pr5* to be master regulators inhibited by beans (activation *z*-scores = −2.530 and −2.535, respectively, *p*-values < 0.0001). Correspondingly, curated by QIAGEN Knowledge Base, the S1P signaling canonical pathway was also inhibited in the bean group (*z*-score = −2.236 and *p*-value = 9.59 × 10^−5^). These results support the metabolite findings showing reduced levels of sphingosines and HCER (Table 6; Figure 8). 

Sphingomyelin hydrolysis is another pathway that affects levels of ceramides in the liver. RNAseq analysis showed that transcription of both sphingomyelin phosphodiesterase 3 (*Smpd3*) and sphingomyelin synthase 1 (*Sgms1*) were downregulated by beans (Table 6). Likewise, QIAGEN’s canonical pathway for sphingolipid metabolism was inhibited with *z*-score = −2.84 and *p*-value = 0.008. *Smpd3* inhibits sphingomyelin production and facilitates the conversion of sphingomyelin to ceramide. This result is consistent with the metabolomics data, showing a significant increase in the levels of various sphingomyelins in the liver of bean-fed mice.

Upstream regulator analysis in IPA provides the opportunity to determine important molecular factors based on the downstream pattern of gene expression from RNAseq datasets. Lipopolysaccharide (LPS) is a significant upstream regulator of *Spt*, and it is markedly inhibited according to upstream regulator predictions from IPA (*z*-score = −8.377, *p*-value = 2.97 × 10^−96^, and *q*-value = 3.27 × 10^−92^). It is a component of the bacterial membrane, indicating that bean suppresses the presence of pathogenic gram-negative bacteria in the gut and thus suppresses LPS-mediated signaling. *Scd* is another upstream regulator of *Spt*, and its expression is lower in beans [18], consistent with a protective phenotype against MASLD [30,31].

Adiponectin, a well-known lipid metabolism regulator, can potentially target hepatic ceramide metabolism by promoting the deacylation of ceramide to sphingosine [32]. IPA analysis predicted upregulation of *Adipoq* (*z*-score = 2.839, *p*-value = 8.22 × 10^−15^, and *q*-value = 3.27 × 10^−13^) based on the downstream DEG patterns. It is possible that the phytochemical profile in beans is stimulating *Adipoq* signaling to confer the reduction of ceramides in the liver. Another significant upstream regulator of bean-induced liver DEGs was S1P (*z*-score = −2.470, *p*-value = 1.36 × 10^−8^, and *q*-value = 2.31 × 10^−7^), whose inhibition indicates its reduced downstream signaling despite insignificant metabolomic data. 

Therefore, transcriptomics data show a pattern of significant suppression of sphingolipid metabolism (de novo synthesis, degradation, and regeneration of ceramides) in the livers of bean-fed animals, further supporting metabolomic profiles thereof. More precise and targeted molecular techniques, however, are necessary to further reveal the key molecular endpoints of bean effects.

### 3.6. Plasma Ceramide Levels for Disease Detection

Ceramide levels are also reduced in the plasma of bean-treated animals compared to controls, as was seen in the liver. Many ceramides and HCER showing differences between treatments in the liver were also different in the plasma (Table 7). These include *N*-palmitoyl-sphingosine (d18:1/16:0) (FC = 0.52, *p*-value = 5.86 × 10^−4^, and *q*-value = 4.36 × 10^−3^), *N*-stearoyl-sphingosine (d18:1/18:0) (FC = 0.24, *p*-value = 1 × 10^−4^, and *q*-value = 9.29 × 10^−4^), ceramide (d18:2/24:1, d18:1/24:2) (FC = 0.55, *p*-value = 7.57 × 10^−5^, and *q*-value = 1.19 × 10^−3^), glycosyl-*N*-palmitoyl-sphingosine (d18:1/16:0) (FC = 0.52, *p*-value = 1.66 × 10^−4^, and *q*-value = 1.66 × 10^−3^), glycosyl-*N*-stearoyl-sphingosine (d18:1/18:0) (FC = 0.33, *p*-value = 1 × 10^−4^, and *q*-value = 1.17 × 10^−3^), glycosyl ceramide (d16:1/24:1, d18:1/22:1) (FC = 0.56, *p*-value = 9.3 × 10^−3^, and *q*-value = 0.037), and glycosyl ceramide (d18:2/24:1, d18:1/24:2) (FC = 0.7, *p*-value = 0.003, and *q*-value = 0.014). Box plots of these metabolites are shown in Supplementary Figure S2. This overlap in results from liver and plasma highlights the potential for using plasma ceramide concentrations as an indicator of ceramide levels in the liver and, therefore, as a biomarker of MASLD risk.

## 4. Discussion

Using a well-documented mouse model for dietary-induced metabolic dysfunction-associated chronic diseases, Figure 1 and Supplementary Figure S1 show that lipid accumulation in the liver is inversely associated with increasing dietary dose of the common bean, i.e., the development of MASLD is inhibited despite the consumption of an obesogenic diet. This effect was observed in both males and females, as previously reported [18,25]. The work reported herein was conceived to use global metabolomic profiling to identify metabolite patterns associated with the prevention of MASLD. To our knowledge, this is the first report that consumption of a diet containing cooked whole bean flour, i.e., beans that are freeze-dried after cooking and milled into a powder, suppresses levels of ceramides in plasma and liver. Accumulation of C16 and C18 ceramides in the liver and elevated levels of these ceramides in plasma are positively associated with insulin resistance and obesity-related diseases, including type 2 diabetes, cardiovascular disease, and cancer [33]. As such, The observed reduction of pathogenic ceramide species in the liver and plasma (Table 1, Table 2, Table 3, Table 4, Table 5, Table 6 and Table 7 and Figure 3, Figure 4, Figure 5, Figure 6 and Figure 7) point to a potential pathway targeted by metabolites of beans directly absorbed from the diet or generated by the gut microbiota and that many account for the many health benefits of consumption of beans and other pulses observed in population studies, but for which evidence remains promising but not definitive [15].

Ceramides are lipid species formed by the condensation of serine and palmitoyl-CoA and are intermediates in the synthesis of complex sphingolipids, such as sphingomyelins and HCER. As such, there is dynamic regulation of cellular ceramide pools through de novo synthesis, biotransformation, and degradation. Under physiological conditions, ceramides support normal cell function through biotransformation to sphingomyelins and hexosylceramides that have structural and regulatory functions. However, certain ceramides, such as C16, C18, and C20, exert pathological activity when they accumulate in the cell. Elevated levels of these ceramides in the liver result in steatosis and lipotoxicity [33]. These ceramides can also interact with pathways involved in insulin resistance, oxidative stress, and inflammation, which are all linked to MASLD [34]. In the early stages of MASLD, ceramides promote hepatic lipid uptake and storage via effects on free fatty acid transporters, such as CD36, and the disruption of protein kinase B (*Akt*) signaling, which inhibits glucose utilization [35,36]. Our findings indicate bean consumption modulates the three main pathways of ceramide homeostasis: de novo synthesis, biotransformation, and degradation based on relative abundances of metabolites and of the gene transcripts that regulate sphingolipid metabolism. Likewise, the dose-dependent effect of bean feeding on the expression of these genes in both male and female mice is consistent with a mechanistic role (Supplementary Figure S4). Overall, the significant reduction of ceramides in both the plasma and liver gives a positive indication of the protective phenotype associated with the consumption of beans. 

The objectives of this study were met in the identification of bean-associated effects on sphingolipid metabolism, given the biological plausibility of a causal role of ceramides in explaining how beans may inhibit the development of MASLD. Recognizing the value of global profiling of metabolites and transcripts in the data-driven investigation that was undertaken while acknowledging the limitations of using the same data for hypothesis testing, it is noteworthy that much of the examination of the pathogenic activity of ceramides has focused on de novo synthesis. De novo ceramide synthesis is regarded as a predominant contributor to pathogenic ceramides, as seen in human liver biopsies of patients with MASLD. Pathogenic dihydroceramides and ceramides, including those of long-chain and very long-chain (16:0, 18:0, 22:0, 23:0, and 24:1), are found in obese adults and at much higher amounts in the livers of MASH patients [34,37]. Two cellular gene targets stand out for having the greatest impact on downstream ceramide synthesis: *Spt* and *Cers6*. These genes are consistently upregulated in both human and animal models of MASH, indicating de novo synthesis as the primary route of ceramide production [3,34]. Increased expression of *CerS6*, an enzyme involved in de novo ceramide synthesis, is known to contribute to MASLD development [33,38], while *Cers6* ablation has recently been shown to inhibit ALD (alcohol-associated liver disease) progression by improving glucose homeostasis [39]. Likewise, liver-specific removal of *Degs1* (dihydroceramide desaturase 1) decreased liver fat mass and improved glucose and insulin tolerance—phenotypes indicative of inhibited MASLD [36]. In fact, inhibitors of de novo ceramide synthesis pathway targets have been tested in preclinical models and shown to diminish the progression of hepatic steatosis [34,40]. However, when levels of transcripts and proteins for these three regulatory nodes (*Sptlc2*, *Cers6*, and *Degs1*) were examined between the groups, differences were numerically small (Supplementary Figures S4–S6). This is not surprising given that these data are from a single snapshot in time that identified a complex metabolic pathway (sphingolipid metabolism) whose multiple components include the synthesis of complex sphingolipids from ceramides as well as regeneration of ceramides from these complex species (sphingomyelins and HCER), and degradation as a potential mechanism of causality. Given that all metabolic processes are implicated by patterns of metabolites and transcripts, the hypothesis-driven extension of this data-driven observation will require quantitative analyses of metabolite concentrations and enzyme activities coupled with kinetic modeling and pathway flux analyses, an example of which is reported in [41,42].

### 4.1. Moving Forward

Dietary patterns containing large amounts of ultra-processed foods, approximately 60% of dietary kilocalories, are highly prevalent in developed and developing countries, along with their associated burden of chronic diseases [43]. However, this has not been the case throughout history. Consumption of a plant-based diet, low in saturated fat and high in disease-preventive phytochemicals, is more consistent with historically observed dietary patterns before the widespread availability and affordability of protein and fat from animal sources [44]. Therefore, as recently proposed by us [45,46,47,48], a bean- and other pulse-rich diet may represent a healthy and affordable alternative to prevalent yet metabolically dysfunctional Western dietary patterns. Cohort and case-control studies in the diet and colon cancer field suggest a minimum dose of 1 to 1.5 cups of cooked pulse per day is necessary for reduced colorectal adenoma risk-benefit. These levels of intake are representative of those studied herein [49,50,51,52,53]. This dose of pulse is much higher than recommended in the 2020–2025 U.S. Dietary Guidelines for Americans, but both the U.S. NHANES and a similar national survey conducted in Canada document that Hispanic Americans and Asian Canadians within these populations consume these high levels of pulses [54,55]. A practical approach to sustaining high levels of pulse consumption is the increased availability to the public of cooked whole food bean (and other pulses) flours. Currently, these flours are commercially produced but are primarily available in pallet lots (500 kg of 20 kg bags). Interestingly, these flours retain >90% of their original dietary protein and fiber and are prepared so that they readily adsorb fluid (pre-gelled), which is an important culinary consideration. A 100 g portion of flour is equivalent to 1.5 cups of cooked beans. The same metrics apply to other pulses.

### 4.2. Strengths and Limitations 

While reporting results from prospective cohort studies or randomized clinical trials is desirable, the data from population studies of health benefits associated with pulse consumption are promising but not definitive. Moreover, to our knowledge, no population-based results address the relationship between dose-dependent pulse consumption and metabolic dysfunction-associated liver diseases in men. Consequently, the use of preclinical studies using a well-defined animal model for MASLD to guide future human studies and investigate knowledge gaps is a strength. An additional strength is the use of a tissue pooling strategy to represent all animals from each dietary group, when tissue was available, from a large common bean dose-response study of long duration in which both biological sexes were investigated (see Section 4.1). Nonetheless, there are limitations: (1) the inability to determine if changes in ceramide metabolism preceded effects on the hepatic accumulation of lipids, (2) the feasibility of having Metabolon evaluate all samples from the dose-response study without sample pooling, and (3) the use of global versus targeted profiling of metabolites and transcripts. However, given the magnitude of the effects observed, a justification now exists for targeted analyses.

## 5. Conclusions

MASLD is characterized by dysregulated lipid metabolism, leading to elevated levels of ceramides. Consumption of cooked common beans protects from MASLD development [18], and this effect is accompanied by the suppression of ceramide accumulation in the liver and in circulating levels of sphingolipid markers of lipotoxicity. Concomitantly, an increase in the health-associated sphingomyelins, which are among the end-products of ceramide metabolic pathways, is observed. RNAseq data revealed patterns of gene expression supporting observed differences in metabolic profiles. Given that high-quality diet and exercise remain first-line interventions in the treatment of metabolic dysfunction-associated liver diseases, pulse-centric dietary strategies, as recently developed by us [45,46,47,48], may provide a foundation for the intersection of precision nutrition with precision medicine to reduce the incidence and mitigate the consequences of metabolic dysfunction resulting from chronic positive energy balance. 

## Figures and Tables

**Table 1:** Metabolites with nominally significant differences between bean-fed intervention and control.

Biospecimen	Effect in Bean vs. Control	Total	Lipid	% Lipid	Sphingolipid-Associated	% Sphingolipid-Associated
Liver	Increased	318	138	43.4	* 9	6.52
	Decreased	169	102	60.36	12	11.76
Plasma	Increased	223	101	45.29	** 5	4.95
	Decreased	173	100	57.8	14	14

Sphingolipid-associated sub-pathways within lipid super-pathway are ceramides, dihydroceramides, hexosylceramides (HCER), sphingomyelins, dihydrosphingomyelins, sphingosines, and sphingolipid synthesis. Metabolites elevated between treatment groups with nominal significance determined by *p* ≤ 0.05. * denotes one and ** denotes two metabolites did not reach *q* < 0.1 significance.

**Table 2:** De novo synthesis of ceramides from liver metabolomics analysis.

Sub Pathway	Biochemical Name	Fold Change	*p*-Value
Glycine, Serine and Threonine Metabolism	serine	**1.74**	0.0281
Fatty Acid Metabolism	palmitoyl CoA	0.77	0.3140
Sphingolipid Synthesis	sphinganine	**0.52**	0.0000
Dihydroceramides	*N*-palmitoyl-sphinganine (d18:0/16:0)	**0.70**	0.0799
Ceramides	*N*-palmitoyl-sphingosine (d18:1/16:0)	**0.54**	0.0003
	*N*-stearoyl-sphingosine (d18:1/18:0) *	**0.31**	0.0000
	*N*-palmitoyl-sphingadienine (d18:2/16:0) *	0.72	0.1610
	*N*-behenoyl-sphingadienine (d18:2/22:0) *	0.89	0.9074
	*N*-palmitoyl-heptadecasphingosine (d17:1/16:0) *	**0.63**	0.0045
	ceramide (d18:1/14:0, d16:1/16:0) *	**0.48**	0.0007
	ceramide (d18:1/17:0, d17:1/18:0) *	0.68	0.1039
	ceramide (d18:2/24:1, d18:1/24:2) *	1.14	0.5097

Welch’s two-sample *t*-test was used to determine nominally significant differences between treatment groups. Fold change with *p* ≤ 0.05 elevations in bean are indicated by red, and reductions in bean are indicated by dark green and light green, respectively. Fold change calculated using means of normalized peak areas. Biochemical name with an asterisk (*) indicates a metabolite that has not been confirmed based on a standard, but Metabolon is confident in its identity.

**Table 3:** Salvage pathway including sphingosines and HCER from liver metabolomics analysis.

Sub Pathway	Biochemical Name	Fold Change	*p*-Value
Sphingosines	sphingosine	**0.57**	0.0000
	hexadecasphingosine (d16:1) *	**0.36**	0.0011
	heptadecasphingosine (d17:1)	**0.55**	0.0011
Hexosylceramides (HCER)	glycosyl-*N*-palmitoyl-sphingosine (d18:1/16:0)	**0.61**	0.0013
	glycosyl-*N*-stearoyl-sphingosine (d18:1/18:0)	**0.29**	0.0005
	glycosyl ceramide (d16:1/24:1, d18:1/22:1) *	**0.33**	0.0000
	glycosyl ceramide (d18:2/24:1, d18:1/24:2) *	0.83	0.6916

Welch’s two-sample *t*-test was used to determine nominally significant differences between treatment groups. Fold change with *p* ≤ 0.05 reductions in bean are indicated by dark green. Fold change calculated using means of normalized peak areas. Biochemical name with an asterisk (*) indicates a metabolite that has not been confirmed based on a standard, but Metabolon is confident in its identity.

**Table 4:** Sphingomyelin biotransformation pathway from liver metabolomics analysis.

Sub Pathway	Biochemical Name	Fold Change	*p*-Value
Dihydrosphingomyelins	palmitoyl dihydrosphingomyelin (d18:0/16:0) *	0.98	0.6848
	behenoyl dihydrosphingomyelin (d18:0/22:0) *	0.90	0.3238
	sphingomyelin (d18:0/18:0, d19:0/17:0) *	0.91	0.2891
	sphingomyelin (d18:0/20:0, d16:0/22:0) *	**0.73**	0.0651
Sphingomyelins	palmitoyl sphingomyelin (d18:1/16:0)	0.94	0.3427
	hydroxypalmitoyl sphingomyelin (d18:1/16:0(OH)) **	0.93	0.4985
	stearoyl sphingomyelin (d18:1/18:0)	0.97	0.8237
	behenoyl sphingomyelin (d18:1/22:0) *	**1.52**	0.0003
	tricosanoyl sphingomyelin (d18:1/23:0) *	1.07	0.3722
	lignoceroyl sphingomyelin (d18:1/24:0)	1.15	0.3174
	sphingomyelin (d18:2/23:1) *	**2.16**	0.0005
	sphingomyelin (d18:1/14:0, d16:1/16:0) *	**0.88**	0.0982
	sphingomyelin (d17:1/16:0, d18:1/15:0, d16:1/17:0) *	1.05	0.3203
	sphingomyelin (d18:2/16:0, d18:1/16:1) *	**1.23**	0.0064
	sphingomyelin (d18:1/17:0, d17:1/18:0, d19:1/16:0)	**1.29**	0.0071
	sphingomyelin (d18:1/18:1, d18:2/18:0)	1.31	0.3226
	sphingomyelin (d18:1/19:0, d19:1/18:0) *	**1.30**	0.0074
	sphingomyelin (d18:1/20:0, d16:1/22:0) *	1.05	0.4648
	sphingomyelin (d18:1/20:1, d18:2/20:0) *	1.21	0.3421
	sphingomyelin (d18:1/21:0, d17:1/22:0, d16:1/23:0) *	**1.75**	0.0007
	sphingomyelin (d18:2/21:0, d16:2/23:0) *	**1.75**	0.0128
	sphingomyelin (d18:1/22:1, d18:2/22:0, d16:1/24:1) *	**1.27**	0.0469
	sphingomyelin (d18:1/22:2, d18:2/22:1, d16:1/24:2) *	**1.87**	0.0006
	sphingomyelin (d18:2/23:0, d18:1/23:1, d17:1/24:1) *	1.05	0.7338
	sphingomyelin (d18:1/24:1, d18:2/24:0) *	0.92	0.3313
	sphingomyelin (d18:2/24:1, d18:1/24:2) *	**1.26**	0.0662

Welch’s two-sample *t*-test was used to determine nominally significant differences between treatment groups. Fold change with *p* ≤ 0.05 and 0.05 < *p* < 0.10 elevations in bean are indicated by red and pink, respectively, and reductions in the bean are indicated by light green. Fold change calculated using means of normalized peak areas. Biochemical name with an asterisk (*) indicates a metabolite that has not been confirmed based on a standard, but Metabolon is confident in its identity. Biochemical name with a double asterisk (**) indicates a metabolite for which a standard is unavailable, but Metabolon is reasonably confident in its identity.

**Table 5:** De novo synthesis of ceramides pathway from liver RNAseq analysis.

Symbol	ID	Entrez Gene Name	Expr Log Ratio	Expr *p*-Value	Expr *q*-Value
*SPTLC1*	ENSMUSG00000021468	serine palmitoyltransferase long chain base subunit 1	−0.1	0.1900	0.4200
*SPTLC2*	ENSMUSG00000021036	serine palmitoyltransferase long chain base subunit 2	**−0.33**	0.0001	0.0014
*SPTLC3*	ENSMUSG00000039092	serine palmitoyltransferase long chain base subunit 3	**2.40**	0.0100	0.0800
*KDSR*	ENSMUSG00000009905	3-ketodihydrosphingosine reductase	**−0.54**	0.0000	0.0000
*CERS1*	ENSMUSG00000087408	ceramide synthase 1	−0.97	0.3400	0.5900
*CERS2*	ENSMUSG00000015714	ceramide synthase 2	0.03	0.6700	0.8300
*CERS3*	ENSMUSG00000030510	ceramide synthase 3	−1.25	0.2000	0.4400
*CERS4*	ENSMUSG00000008206	ceramide synthase 4	−0.09	0.6900	0.8500
*CERS5*	ENSMUSG00000023021	ceramide synthase 5	−0.03	0.9100	0.9600
*CERS6*	ENSMUSG00000027035	ceramide synthase 6	**−0.44**	0.0005	0.0062
*DEGS1*	ENSMUSG00000038633	delta 4-desaturase, sphingolipid 1	0.06	0.4800	0.7100
*DEGS2*	ENSMUSG00000021263	delta 4-desaturase, sphingolipid 2	−0.4	0.5200	0.7400

Expression log ratio with *p* ≤ 0.05 elevations in bean are indicated by red, and reductions in bean are indicated by dark green.

**Table 6:** Degradation and biotransformation of ceramides pathway from liver RNAseq analysis.

Symbol	ID	Entrez Gene Name	Expr Log Ratio	Expr *p*-Value	Expr *q*-Value
*ACER2*	ENSMUSG00000038007	alkaline ceramidase 2	**−0.38**	0.0034	0.0300
*ASAH1*	ENSMUSG00000031591	N-acylsphingosine amidohydrolase 1	**−0.16**	0.0400	0.1500
*ASAH2*	ENSMUSG00000024887	N-acylsphingosine amidohydrolase 2	**−0.30**	0.0005	0.0061
*CERK*	ENSMUSG00000035891	ceramide kinase	**−0.39**	0.0300	0.1400
*CERT1*	ENSMUSG00000021669	ceramide transporter 1	**−0.31**	0.0000	0.0006
*PSAP*	ENSMUSG00000004207	prosaposin	**−0.23**	0.0200	0.1000
*SGPP1*	ENSMUSG00000021054	sphingosine-1-phosphate phosphatase 1	**0.24**	0.0008	0.0090
*SGSM1*	ENSMUSG00000042216	small G protein signaling modulator 1	**−0.42**	0.0200	0.1000
*SMPD3*	ENSMUSG00000031906	sphingomyelin phosphodiesterase 3	**−2.70**	0.0000	0.0000
*UGCG*	ENSMUSG00000028381	UDP-glucose ceramide glucosyltransferase	**−0.25**	0.0200	0.0900

Expression log ratio with *p* ≤ 0.05 elevations in bean are indicated by red, and reductions in bean are indicated by dark green.

**Table 7:** Ceramides measured in plasma by metabolomics analysis.

Sub Pathway	Biochemical Name	Fold Change	*p*-Value
Ceramides	*N*-palmitoyl-sphingosine (d18:1/16:0)	**0.52**	0.0006
	*N*-stearoyl-sphingosine (d18:1/18:0) *	**0.24**	0.0001
	ceramide (d18:1/14:0, d16:1/16:0) *	**0.52**	0.0597
	ceramide (d18:1/20:0, d16:1/22:0, d20:1/18:0)*	**0.51**	0.0136
	ceramide (d16:1/24:1, d18:1/22:1) *	2.23	0.1209
	ceramide (d18:2/24:1, d18:1/24:2) *	**0.55**	0.0001
Hexosylceramides (HCER)	glycosyl-*N*-palmitoyl-sphingosine (d18:1/16:0)	**0.52**	0.0002
	glycosyl-*N*-stearoyl-sphingosine (d18:1/18:0)	**0.33**	0.0001
	glycosyl-*N*-behenoyl-sphingosine (d18:1/22:0) *	1.04	0.9564
	glycosyl-*N*-nervonoyl-sphingosine (d18:1/24:1) *	0.57	0.1257
	glycosyl ceramide (d18:1/20:0, d16:1/22:0) *	0.79	0.5237
	glycosyl ceramide (d16:1/24:1, d18:1/22:1) *	**0.56**	0.0093
	glycosyl ceramide (d18:1/23:1, d17:1/24:1) *	**0.43**	0.0720
	glycosyl ceramide (d18:2/24:1, d18:1/24:2) *	**0.70**	0.0026
Lactosylceramides (LCER)	lactosyl-*N*-palmitoyl-sphingosine (d18:1/16:0)	**0.67**	0.0101

Welch’s two-sample *t*-test was used to determine nominally significant differences between treatment groups. Fold change with *p* ≤ 0.05 and 0.05 < *p* < 0.10 reductions in bean are indicated by dark green and light green, respectively. Fold change calculated using means of normalized peak areas. Biochemical name with an asterisk (*) indicates a metabolite that has not been confirmed based on a standard, but Metabolon is confident in its identity.

## Data Availability

The RNA sequencing data reported herein is submitted to the Gene Expression Omnibus, a database for gene expression profiling managed by the National Center for Biotechnology Information. Please contact the corresponding author for the GEO accession number.

## Supplementary Materials

The following supporting information can be downloaded at: https://www.mdpi.com/article/10.3390/nu16183196/s1, Table S1: Study design. Table S2: Diet formulations. Figure S1. Anti-steatotic effect of bean in the liver of female mice. Figure S2: Box plots of selected ceramide metabolites from plasma. Figure S3: Patterns of change in ceramide-related metabolites in males and females in liver and plasma samples. Figure S4. Box plots of genes expressed in the ceramide production from the de novo biosynthesis, salvage, and sphingomyelin hydrolysis pathways between females and males. Figure S5. Box plots of protein levels in the ceramide de novo biosynthesis pathway in males. Figure S6. Western blot gel images of protein levels in the ceramide de novo biosynthesis pathway in the liver.

## References

[1] Chan W.K., Chuah K.H., Rajaram R.B., Lim L.L., Ratnasingam J., Vethakkan S.R. (2023). Metabolic Dysfunction-Associated Steatotic Liver Disease (MASLD): A State-of-the-Art Review. J. Obes. Metab. Syndr..

[2] Jiang Y., Wu L., Zhu X., Bian H., Gao X., Xia M. (2024). Advances in management of metabolic dysfunction-associated steatotic liver disease: From mechanisms to therapeutics. Lipids Health Dis..

[3] Poss A.M., Summers S.A. (2020). Too Much of a Good Thing? An Evolutionary Theory to Explain the Role of Ceramides in NAFLD. Front. Endocrinol..

[4] Gu S., Qiao Y., Liu S., Yang S., Cong S., Wang S., Yu D., Wang W., Chai X. (2023). Frontiers and hotspots of adipose tissue and NAFLD: A bibliometric analysis from 2002 to 2022. Front. Physiol..

[5] Khanmohammadi S., Tavolinejad H., Aminorroaya A., Rezaie Y., Ashraf H., Vasheghani-Farahani A. (2022). Association of lipid accumulation product with type 2 diabetes mellitus, hypertension, and mortality: A systematic review and meta-analysis. J. Diabetes Metab. Disord..

[6] Miao L., Targher G., Byrne C.D., Cao Y.-Y., Zheng M.-H. (2024). Current status and future trends of the global burden of MASLD. Trends Endocrinol. Metab..

[7] Yanai H., Adachi H., Hakoshima M., Iida S., Katsuyama H. (2023). Metabolic-Dysfunction-Associated Steatotic Liver Disease-Its Pathophysiology, Association with Atherosclerosis and Cardiovascular Disease, and Treatments. Int. J. Mol. Sci..

[8] Harrison S.A., Bedossa P., Guy C.D., Schattenberg J.M., Loomba R., Taub R., Labriola D., Moussa S.E., Neff G.W., Rinella M.E. (2024). A Phase 3, Randomized, Controlled Trial of Resmetirom in NASH with Liver Fibrosis. N. Engl. J. Med..

[9] Younossi Z.M., Corey K.E., Lim J.K. (2021). AGA Clinical Practice Update on Lifestyle Modification Using Diet and Exercise to Achieve Weight Loss in the Management of Nonalcoholic Fatty Liver Disease: Expert Review. Gastroenterology.

[10] Hydes T.J., Ravi S., Loomba R., Gray M.E. (2020). Evidence-based clinical advice for nutrition and dietary weight loss strategies for the management of NAFLD and NASH. Clin. Mol. Hepatol..

[11] Abenavoli L., Milic N. (2013). Dietary intervention in non-alcoholic fatty liver disease. J. Acad. Nutr. Diet..

[12] Stefano J.T., Duarte S.M.B., Ribeiro Leite Altikes R.G., Oliveira C.P. (2023). Non-pharmacological management options for MAFLD: A practical guide. Ther. Adv. Endocrinol. Metab..

[13] Zhu Y., Yang H., Zhang Y., Rao S., Mo Y., Zhang H., Liang S., Zhang Z., Yang W. (2022). Dietary fiber intake and non-alcoholic fatty liver disease: The mediating role of obesity. Front. Public Health.

[14] Rodriguez L., Mendez D., Montecino H., Carrasco B., Arevalo B., Palomo I., Fuentes E. (2022). Role of Phaseolus vulgaris L. in the Prevention of Cardiovascular Diseases-Cardioprotective Potential of Bioactive Compounds. Plants.

[15] Didinger C., Thompson H.J. (2022). The role of pulses in improving human health: A review. Legume Sci..

[16] Zhao L., Jin L., Petrick J.L., Zeng H., Wang F., Tang L., Smith-Warner S.A., Eliassen A.H., Zhang F.F., Campbell P.T. (2023). Specific botanical groups of fruit and vegetable consumption and liver cancer and chronic liver disease mortality: A prospective cohort study. Am. J. Clin. Nutr..

[17] Tucker L.A. (2023). Legume Intake, Body Weight, and Abdominal Adiposity: 10-Year Weight Change and Cross-Sectional Results in 15,185 U.S. Adults. Nutrients.

[18] Lutsiv T., McGinley J.N., Neil E.S., Foster M.T., Thompson H.J. (2023). Thwarting Metabolic Dysfunction-Associated Fatty Liver Disease (MAFLD) with Common Bean: Dose- and Sex-Dependent Protection against Hepatic Steatosis. Nutrients.

[19] Lutsiv T., McGinley J.N., Neil-McDonald E.S., Weir T.L., Foster M.T., Thompson H.J. (2022). Relandscaping the Gut Microbiota with a Whole Food: Dose-Response Effects to Common Bean. Foods.

[20] Thompson H.J., McGinley J.N., Neil E.S., Brick M.A. (2017). Beneficial Effects of Common Bean on Adiposity and Lipid Metabolism. Nutrients.

[21] Nagral A., Bangar M., Menezes S., Bhatia S., Butt N., Ghosh J., Manchanayake J.H., Al Mahtab M., Singh S.P. (2022). Gender Differences in Nonalcoholic Fatty Liver Disease. Euroasian J. Hepatogastroenterol..

[22] Moran-Costoya A., Proenza A.M., Gianotti M., Llado I., Valle A. (2021). Sex Differences in Nonalcoholic Fatty Liver Disease: Estrogen Influence on the Liver-Adipose Tissue Crosstalk. Antioxid. Redox Signal..

[23] U.S. Department of Agriculture and U.S. Department of Health and Human Services Dietary Guidelines for Americans, 2020–2025. 9th Edition. December 2020. https://www.dietaryguidelines.gov.

[24] Blaise B.J., Correia G., Tin A., Young J.H., Vergnaud A.-C., Lewis M., Pearce J.T.M., Elliott P., Nicholson J.K., Holmes E. (2016). Power Analysis and Sample Size Determination in Metabolic Phenotyping. Anal. Chem..

[25] Chu D.T., Malinowska E., Jura M., Kozak L.P. (2017). C57BL/6J mice as a polygenic developmental model of diet-induced obesity. Physiol. Rep..

[26] Ford L., Kennedy A.D., Goodman K.D., Pappan K.L., Evans A.M., Miller L.A.D., Wulff J.E., Wiggs B.R., Lennon J.J., Elsea S. (2020). Precision of a Clinical Metabolomics Profiling Platform for Use in the Identification of Inborn Errors of Metabolism. J. Appl. Lab. Med..

[27] Walker J.M., Walker J.M. (1994). The Bicinchoninic Acid (BCA) Assay for Protein Quantitation. Basic Protein and Peptide Protocols.

[28] Thompson H.J., Lutsiv T., McGinley J.N., Fitzgerald V.K., Neil E.S. (2023). Consumption of Common Bean Suppresses the Obesogenic Increase in Adipose Depot Mass: Impact of Dose and Biological Sex. Nutrients.

[29] Benjamini Y., Hochberg Y. (1995). Controlling the false discovery rate: A practical and powerful approach to multiple testing. JR Stat. Soc. B.

[30] Esler W.P., Cohen D.E. (2024). Pharmacologic inhibition of lipogenesis for the treatment of NAFLD. J. Hepatol..

[31] Johnson E.L., Heaver S.L., Waters J.L., Kim B.I., Bretin A., Goodman A.L., Gewirtz A.T., Worgall T.S., Ley R.E. (2020). Sphingolipids produced by gut bacteria enter host metabolic pathways impacting ceramide levels. Nat. Commun..

[32] Wilkerson J.L., Tatum S.M., Holland W.L., Summers S.A. (2024). Ceramides are fuel gauges on the drive to cardiometabolic disease. Physiol. Rev..

[33] Turpin-Nolan S.M., Bruning J.C. (2020). The role of ceramides in metabolic disorders: When size and localization matters. Nat. Rev. Endocrinol..

[34] Yu X.D., Wang J.W. (2022). Ceramide de novo synthesis in non-alcoholic fatty liver disease: Pathogenic mechanisms and therapeutic perspectives. Biochem. Pharmacol..

[35] Hajduch E., Lachkar F., Ferre P., Foufelle F. (2021). Roles of Ceramides in Non-Alcoholic Fatty Liver Disease. J. Clin. Med..

[36] Chaurasia B., Tippetts T.S., Mayoral Monibas R., Liu J., Li Y., Wang L., Wilkerson J.L., Sweeney C.R., Pereira R.F., Sumida D.H. (2019). Targeting a ceramide double bond improves insulin resistance and hepatic steatosis. Science.

[37] Luukkonen P.K., Zhou Y., Sadevirta S., Leivonen M., Arola J., Oresic M., Hyotylainen T., Yki-Jarvinen H. (2016). Hepatic ceramides dissociate steatosis and insulin resistance in patients with non-alcoholic fatty liver disease. J. Hepatol..

[38] Xia J.Y., Holland W.L., Kusminski C.M., Sun K., Sharma A.X., Pearson M.J., Sifuentes A.J., McDonald J.G., Gordillo R., Scherer P.E. (2015). Targeted Induction of Ceramide Degradation Leads to Improved Systemic Metabolism and Reduced Hepatic Steatosis. Cell Metab..

[39] Jeon S., Scorletti E., Dempsey J., Buyco D., Lin C., Saiman Y., Bayen S., Harkin J., Martin J., Hooks R. (2023). Ceramide synthase 6 (CerS6) is upregulated in alcohol-associated liver disease and exhibits sex-based differences in the regulation of energy homeostasis and lipid droplet accumulation. Mol. Metab..

[40] Jiang M., Li C., Liu Q., Wang A., Lei M. (2019). Inhibiting Ceramide Synthesis Attenuates Hepatic Steatosis and Fibrosis in Rats with Non-alcoholic Fatty Liver Disease. Front. Endocrinol..

[41] Wronowska W., Charzyńska A., Nienałtowski K., Gambin A. (2015). Computational modeling of sphingolipid metabolism. BMC Syst. Biol..

[42] Canals D., Clarke C.J. (2022). Compartmentalization of Sphingolipid metabolism: Implications for signaling and therapy. Pharmacol. Ther..

[43] Nees S., Lutsiv T., Thompson H.J. (2024). Ultra-Processed Foods-Dietary Foe or Potential Ally?. Nutrients.

[44] Abe-Inge V., Aidoo R., Moncada de la Fuente M., Kwofie E.M. (2024). Plant-based dietary shift: Current trends, barriers, and carriers. Trends Food Sci. Technol..

[45] Didinger C., Bunning M., Thompson H. (2023). A Translational Approach to Increase Pulse Intake and Promote Public Health through Developing an Extension Bean Toolkit. Nutrients.

[46] Didinger C., Bunning M., Thompson H.J. (2023). Bean Cuisine: The Potential of Citizen Science to Help Motivate Changes in Pulse Knowledge and Consumption. Foods.

[47] Didinger C., Thompson H. (2020). Motivating Pulse-Centric Eating Patterns to Benefit Human and Environmental Well-Being. Nutrients.

[48] Didinger C., Thompson H.J. (2021). Defining Nutritional and Functional Niches of Legumes: A Call for Clarity to Distinguish a Future Role for Pulses in the Dietary Guidelines for Americans. Nutrients.

[49] Lanza E., Hartman T.J., Albert P.S., Shields R., Slattery M., Caan B., Paskett E., Iber F., Kikendall J.W., Lance P. (2006). High dry bean intake and reduced risk of advanced colorectal adenoma recurrence among participants in the polyp prevention trial. J. Nutr..

[50] Wang Y., Wang Z., Fu L., Chen Y., Fang J. (2013). Legume consumption and colorectal adenoma risk: A meta-analysis of observational studies. PLoS ONE.

[51] Agurs-Collins T., Smoot D., Afful J., Makambi K., Adams-Campbell L.L. (2006). Legume intake and reduced colorectal adenoma risk in African-Americans. J. Natl. Black Nurses Assoc..

[52] Patel L., La Vecchia C., Negri E., Mignozzi S., Augustin L.S.A., Levi F., Serraino D., Giacosa A., Alicandro G. (2024). Legume intake and cancer risk in a network of case-control studies. Eur. J. Clin. Nutr..

[53] Zhang X., Irajizad E., Hoffman K.L., Fahrmann J.F., Li F., Seo Y.D., Browman G.J., Dennison J.B., Vykoukal J., Luna P.N. (2023). Modulating a prebiotic food source influences inflammation and immune-regulating gut microbes and metabolites: Insights from the BE GONE trial. eBioMedicine.

[54] Mitchell D.C., Lawrence F.R., Hartman T.J., Curran J.M. (2009). Consumption of dry beans, peas, and lentils could improve diet quality in the US population. J. Am. Diet. Assoc..

[55] Mudryj A.N., Yu N., Hartman T.J., Mitchell D.C., Lawrence F.R., Aukema H.M. (2012). Pulse consumption in Canadian adults influences nutrient intakes. Br. J. Nutr..

