# Supplementary material for: Common Bean Suppresses Hepatic Ceramide Metabolism in a Mouse Model of Metabolic Dysfunction-Associated Steatotic Liver Disease
Source: Nutrients. 2024 Sep 21;16(18):3196. doi: 10.3390/nu16183196 (PMC11434909; doi:10.3390/nu16183196)
Supplement: Supplementary file 1 [file nutrients-16-03196-s001.zip › Supplementary Figures.pdf]

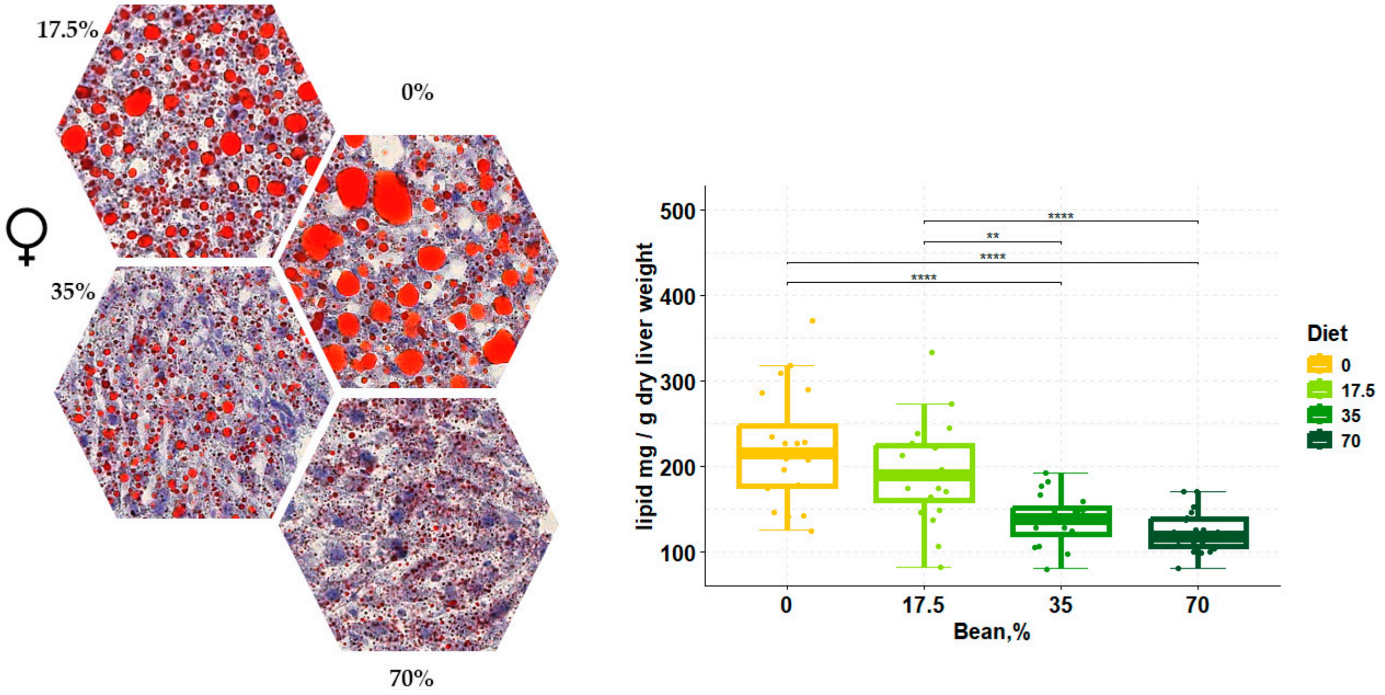

(**a**) (**b**)

Figure S1. Bean prevents the accumulation of lipid droplets in the liver of female animals in a dose-dependent manner. (a) Oil Red O- and H&E-stained liver sections across different diets: percentage indicates the amount of total dietary protein derived from common bean. (b) Box plots of the bean dose effects on the hepatic lipid. Values represent amount of lipid in mg normalized to g of dry liver weight across the diet groups. Groups indicate total dietary protein percent sourced by bean. Kruskal–Wallis testing showed significant differences by the diet effect ($\chi^2 = 37.78$, p-value = 3.147×10^{-8}) with the large effect size ($\eta^2 = 0.458$). Pairwise comparisons between the diet group were conducted using the *post-hoc* Dunn test: ** q-value < 0.01; *** q-value < 0.001; **** q-value < 0.0001.

Ceramides

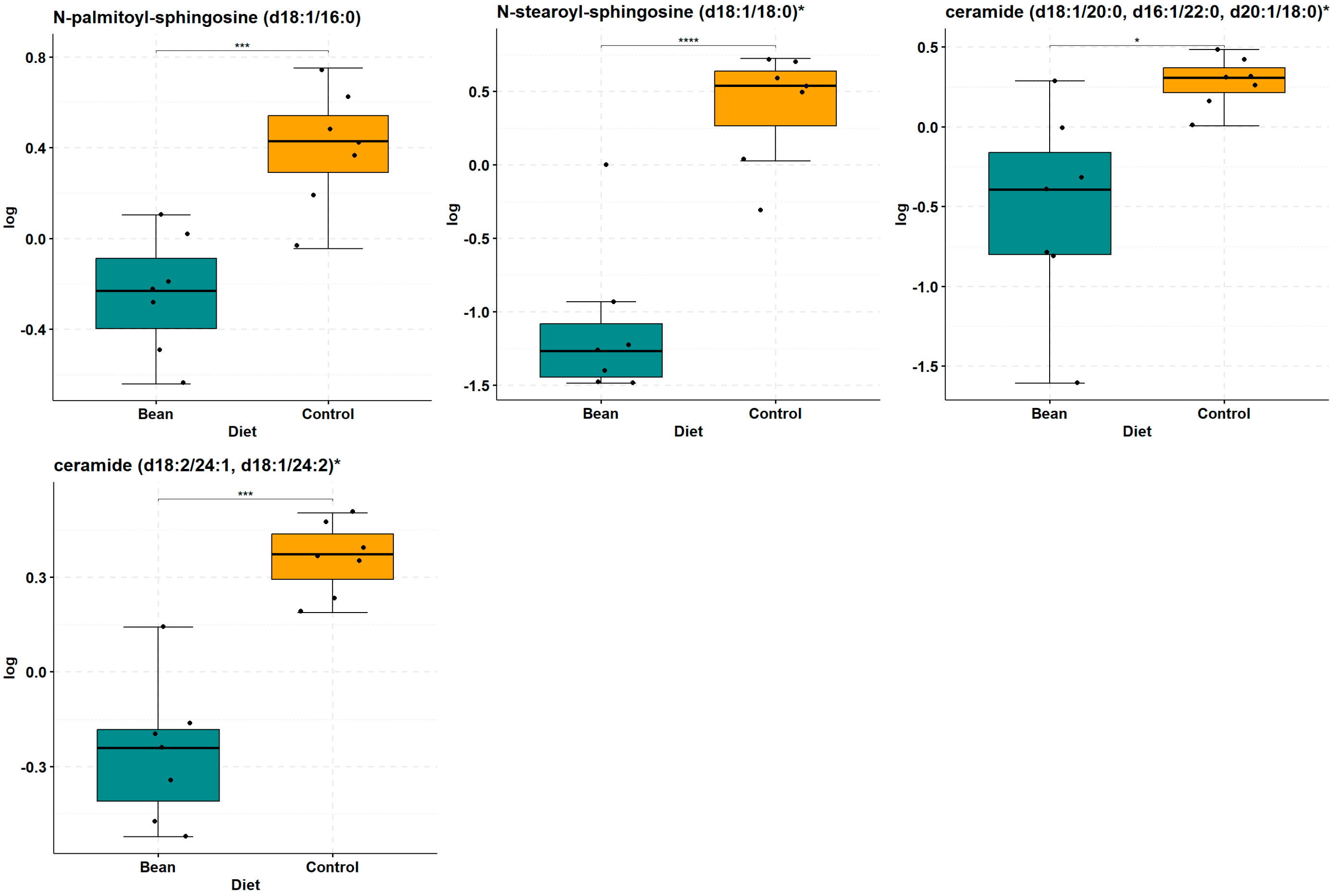

N-palmitoyl-sphingosine (d18:1/16:0)
***
0.8
0.4
0.0
-0.4
log
Bean
Control
Diet
N-stearoyl-sphingosine (d18:1/18:0)*
****
0.5
0.0
-0.5
-1.0
-1.5
log
Bean
Control
Diet
ceramide (d18:1/20:0, d16:1/22:0, d20:1/18:0)*
*
0.5
0.0
-0.5
-1.0
-1.5
log
Bean
Control
Diet
ceramide (d18:2/24:1, d18:1/24:2)*
***
0.3
0.0
-0.3
log
Bean
Control
Diet

Hexosyl- and lactosylceramides

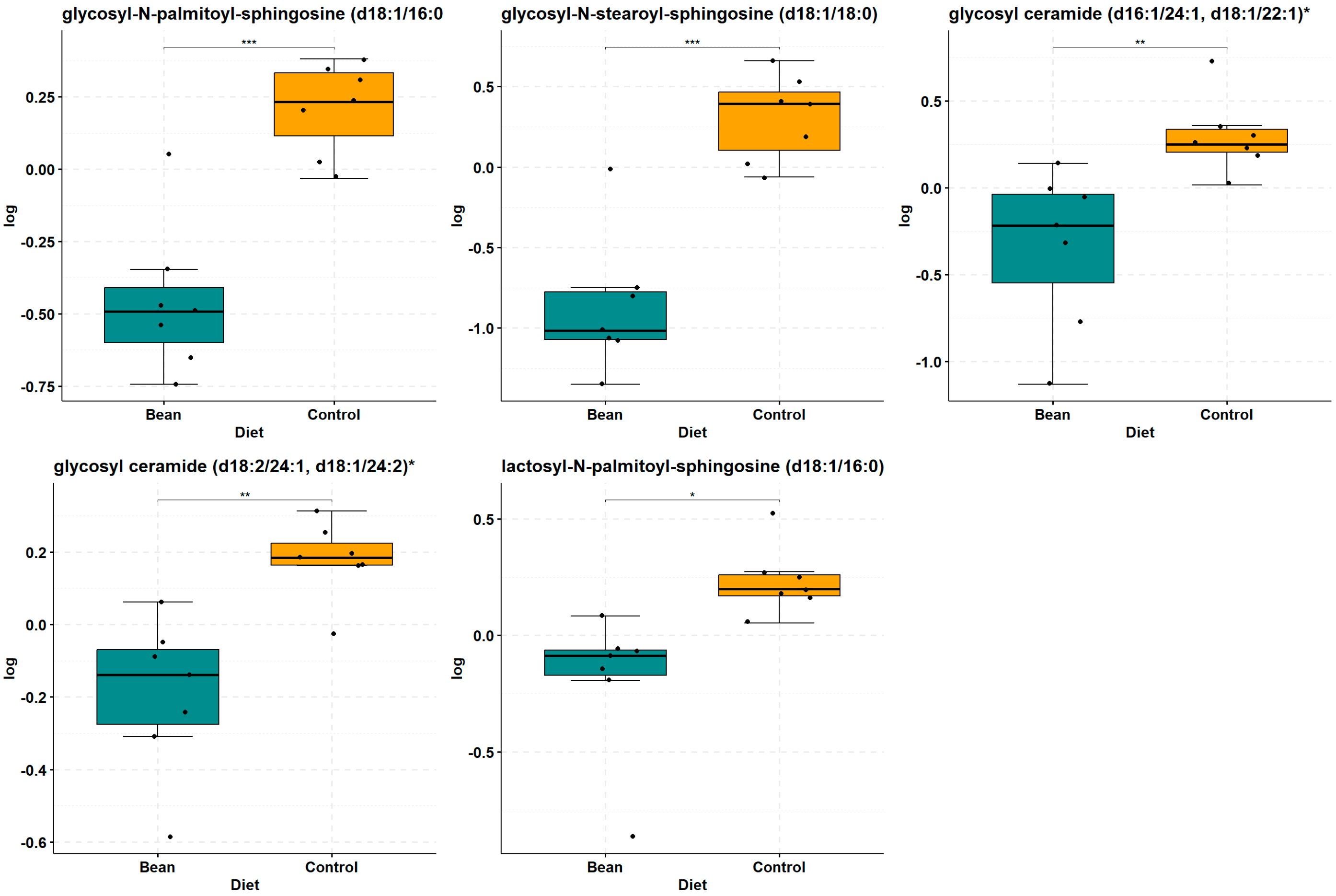

Figure S2. Box plots of selected ceramide metabolites from the plasma. Statistically significant differences where * q-value < 0.05; ** q-value < 0.01; *** q-value < 0.001; **** q-value < 0.0001.

De novo ceramide synthesis substrates

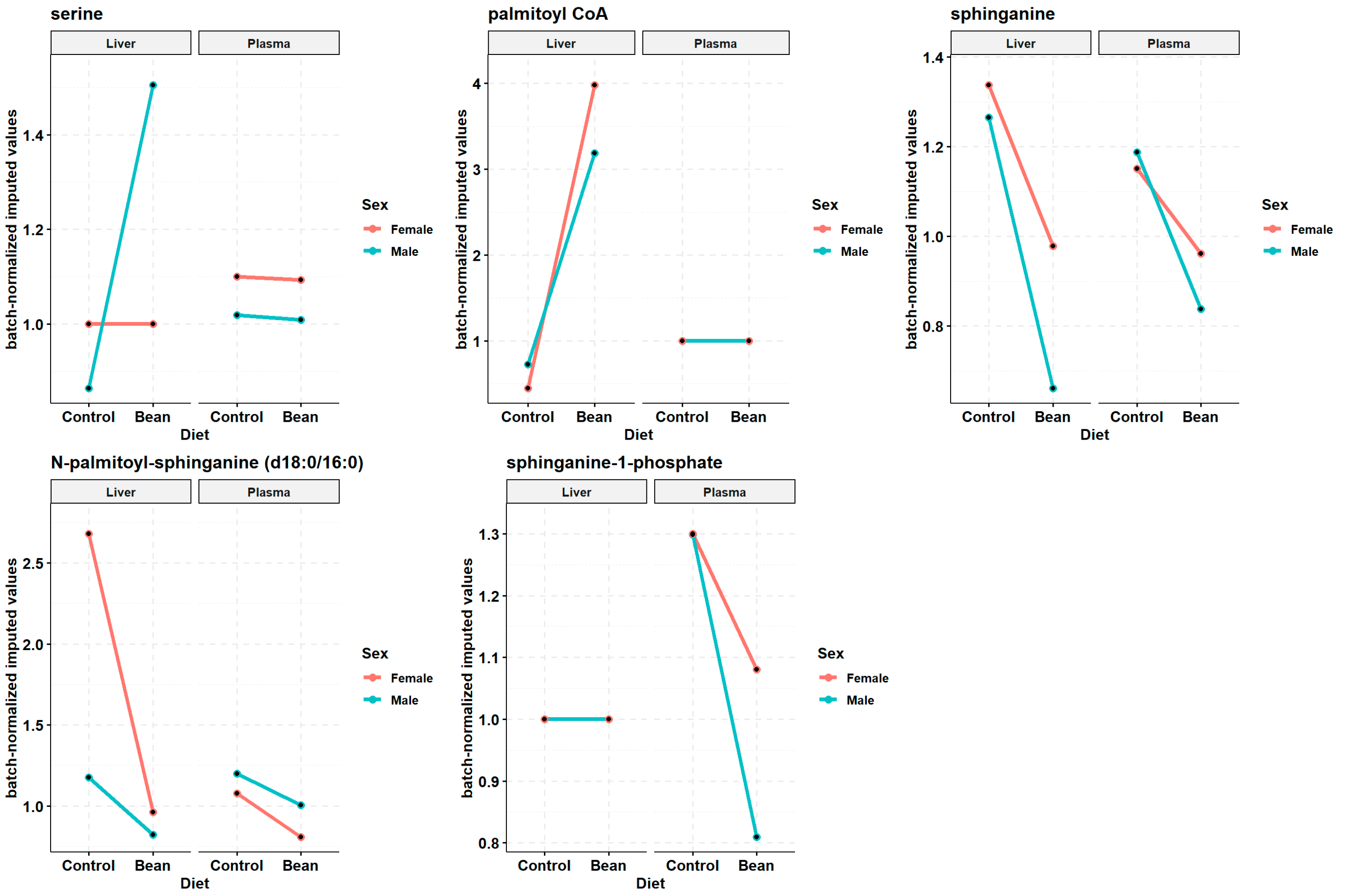

Ceramides

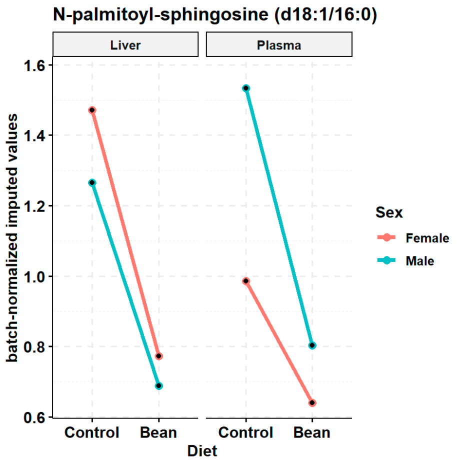
N-palmitoyl-sphingosine (d18:1/16:0)
Liver
Plasma
batch-normalized imputed values
Sex
Female
Male
Control
Bean
Diet

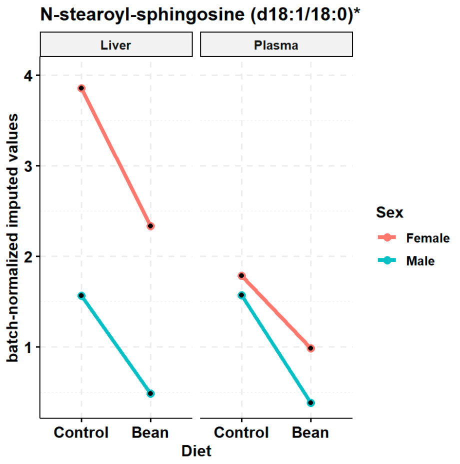
N-stearoyl-sphingosine (d18:1/18:0)*
Liver
Plasma
batch-normalized imputed values
Sex
Female
Male
Control
Bean
Diet

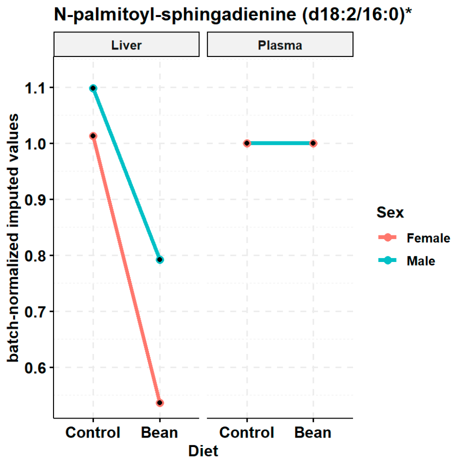
N-palmitoyl-sphingadienine (d18:2/16:0)*
Liver
Plasma
batch-normalized imputed values
Sex
Female
Male
Control
Bean
Diet

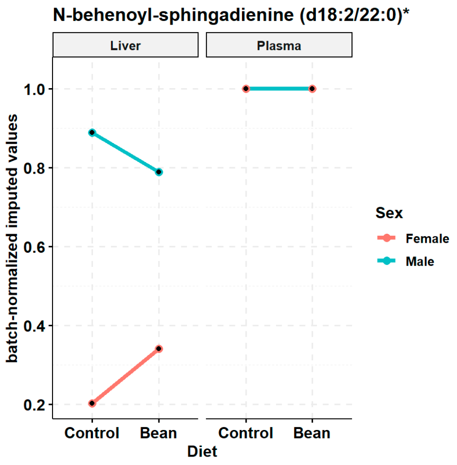
N-behenoyl-sphingadienine (d18:2/22:0)*
Liver
Plasma
batch-normalized imputed values
Sex
Female
Male
Control
Bean
Diet

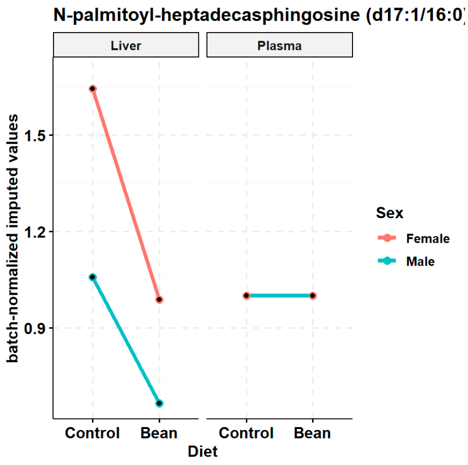
N-palmitoyl-heptadecasphingosine (d17:1/16:0)
Liver
Plasma
batch-normalized imputed values
Sex
Female
Male
Control
Bean
Diet

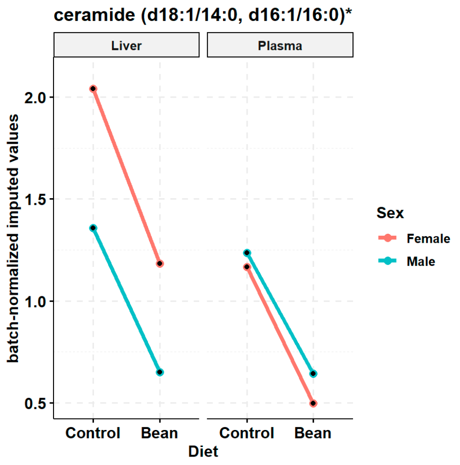
ceramide (d18:1/14:0, d16:1/16:0)*
Liver
Plasma
batch-normalized imputed values
Sex
Female
Male
Control
Bean
Diet

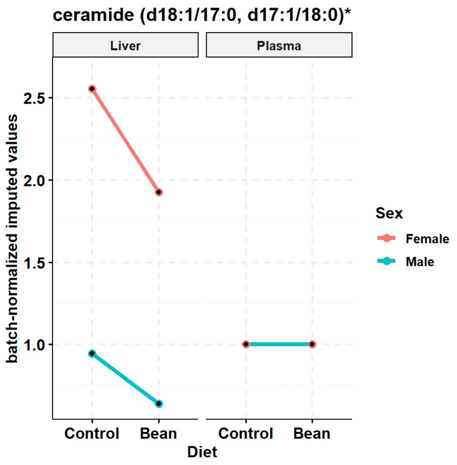
ceramide (d18:1/17:0, d17:1/18:0)*
Liver
Plasma
batch-normalized imputed values
Sex
Female
Male
Control
Bean
Diet

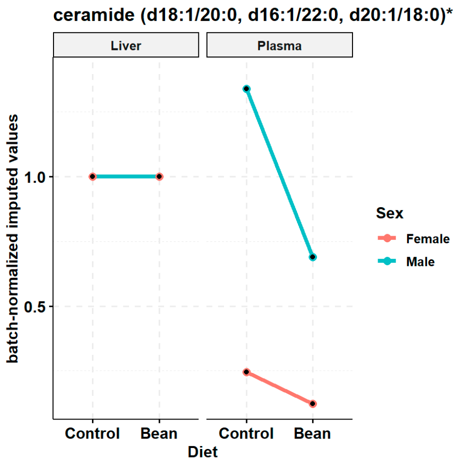
ceramide (d18:1/20:0, d16:1/22:0, d20:1/18:0)*
Liver
Plasma
batch-normalized imputed values
Sex
Female
Male
Control
Bean
Diet

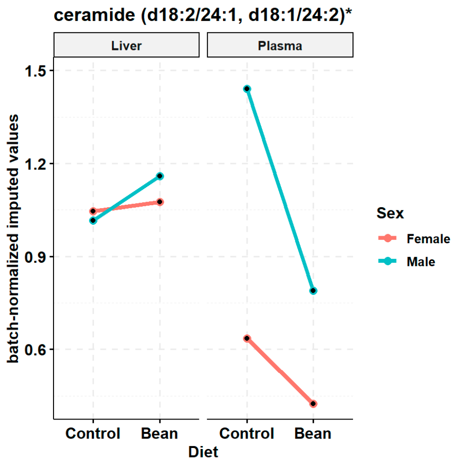
ceramide (d18:2/24:1, d18:1/24:2)*
Liver
Plasma
batch-normalized imputed values
Sex
Female
Male
Control
Bean
Diet

Sphingosines

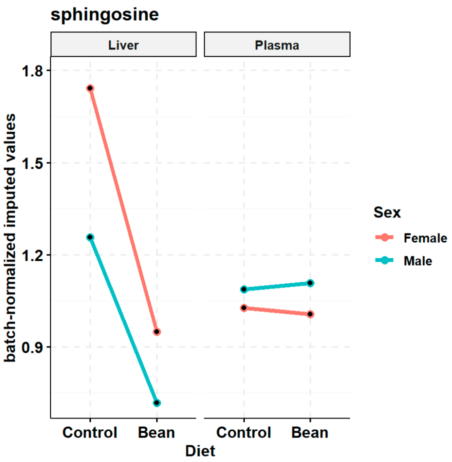

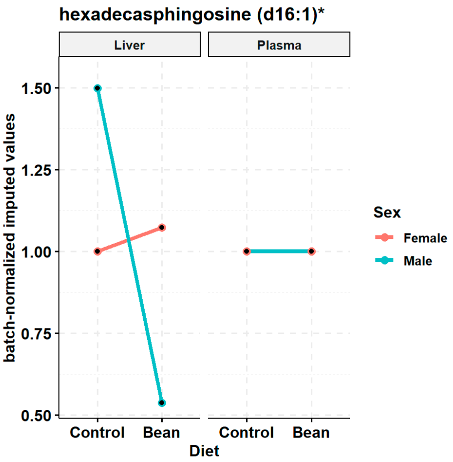

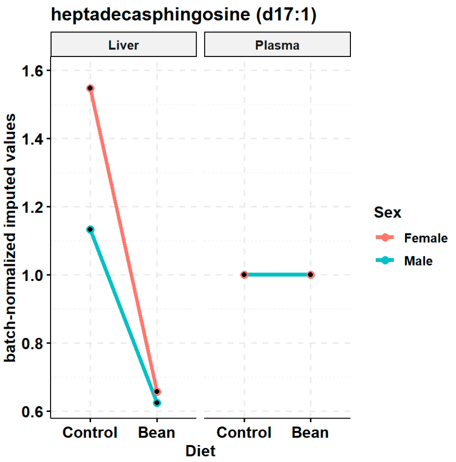

Hexosyl- and lactosylceramides

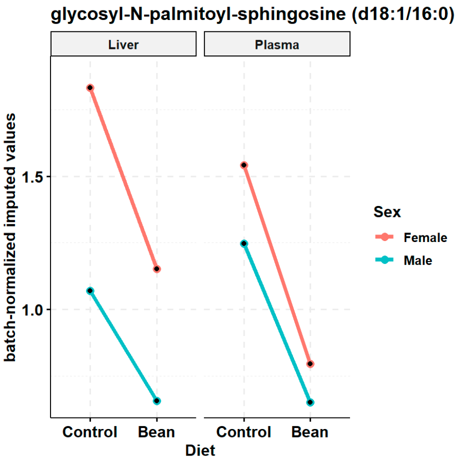

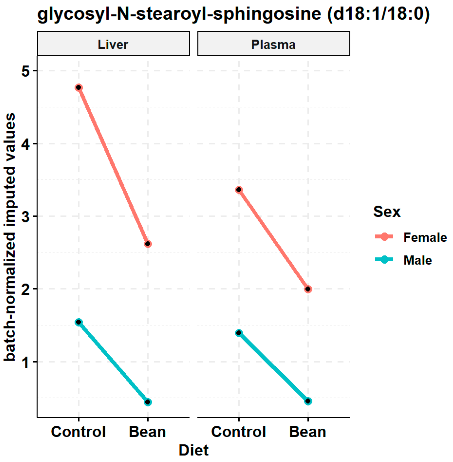

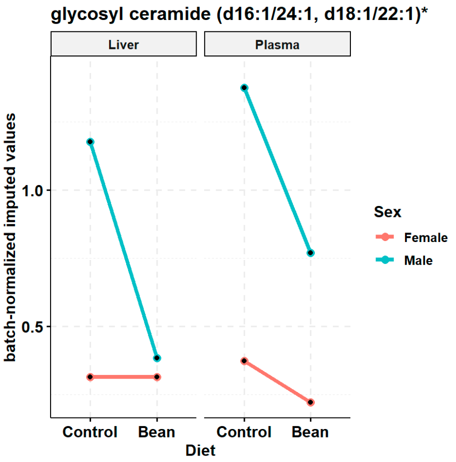

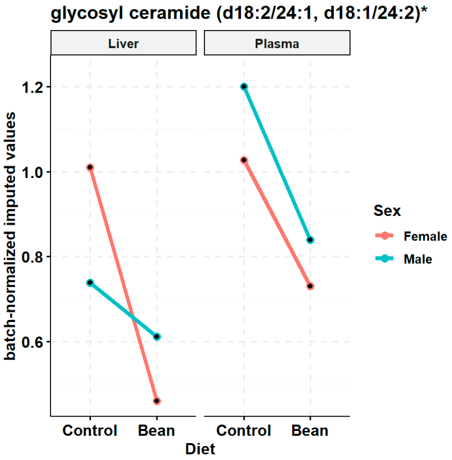

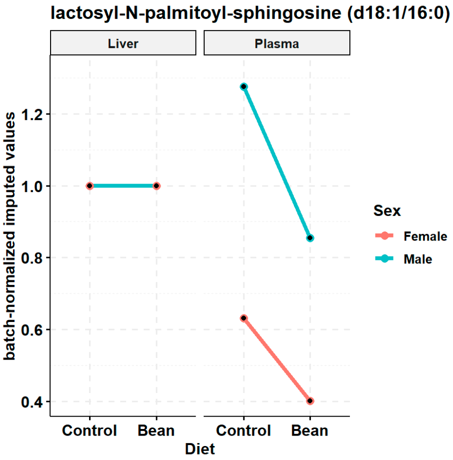

Sphingomyelins

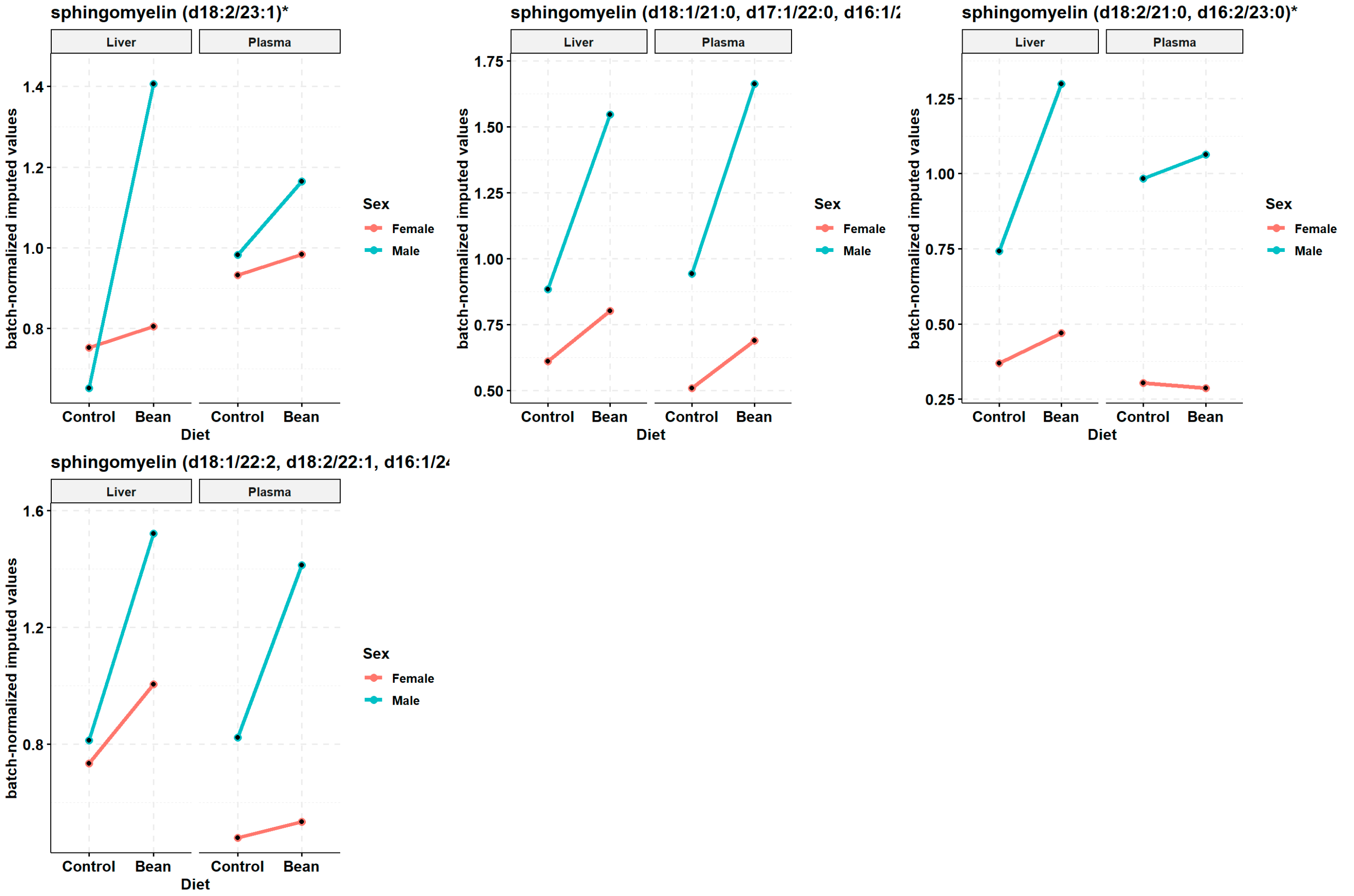

Figure S3. Patterns of change in ceramide-related metabolites in males and females in liver and plasma samples. Dots indicate averaged values of metabolite abundance.

Ceramide *de novo* biosynthesis pathway

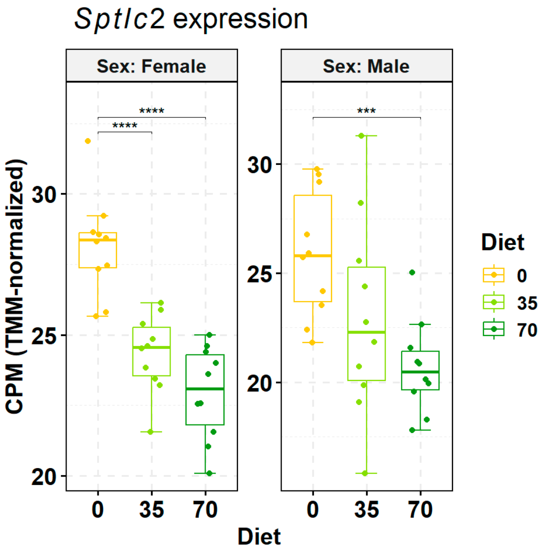

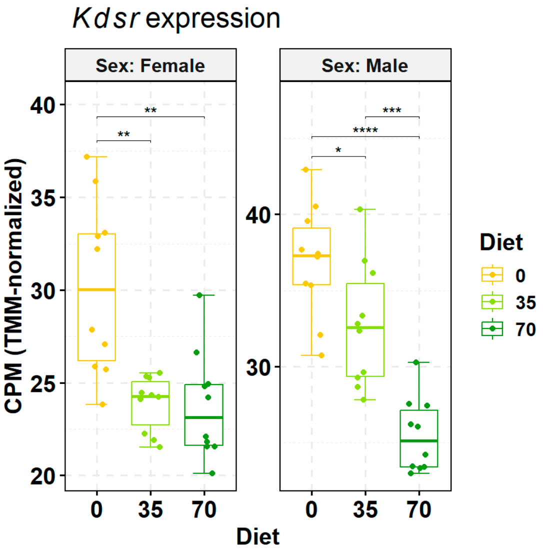

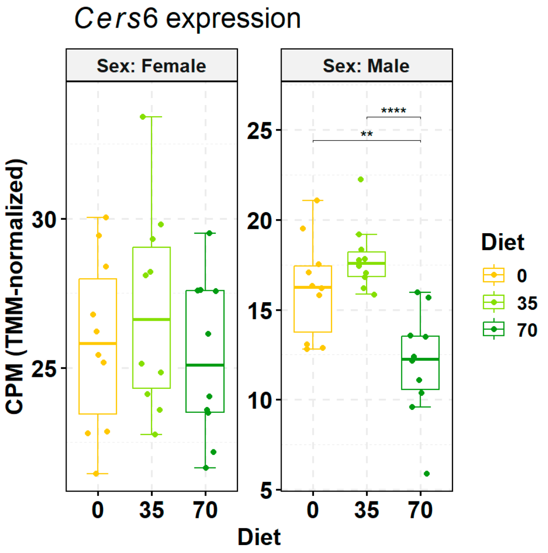

<u>Ceramides biotransformation and degradation pathways</u>

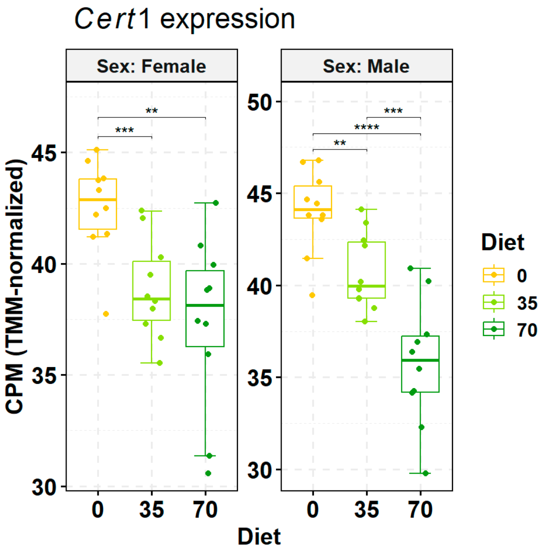

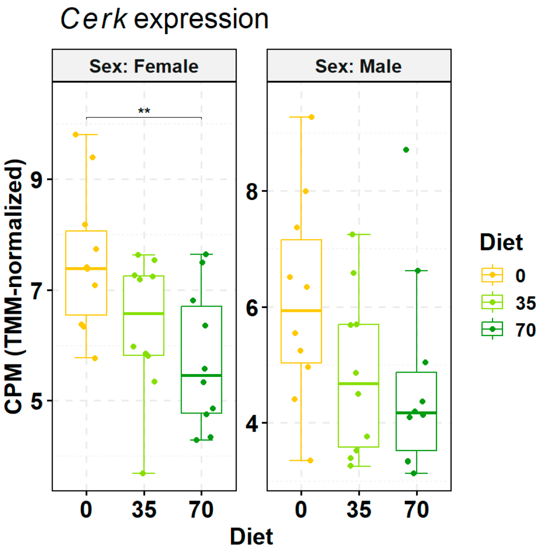

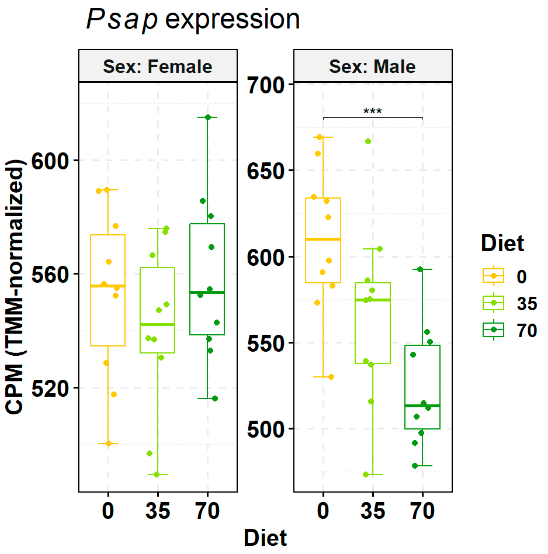

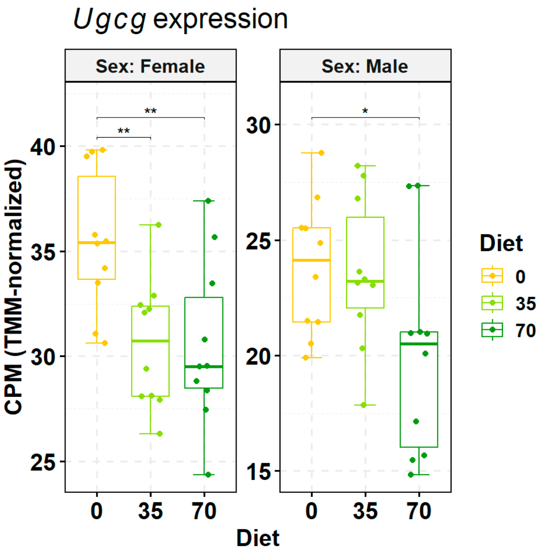

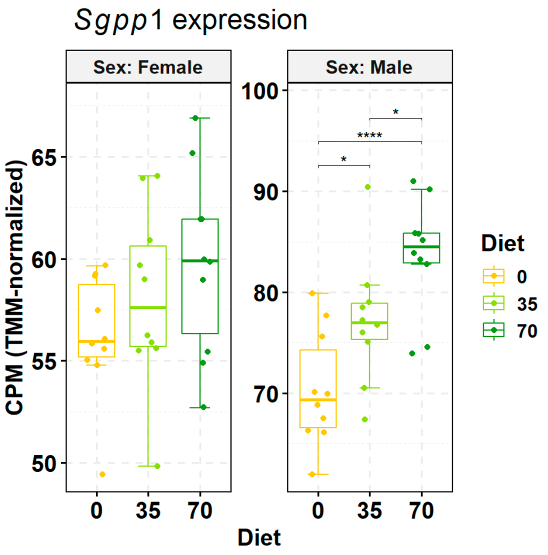

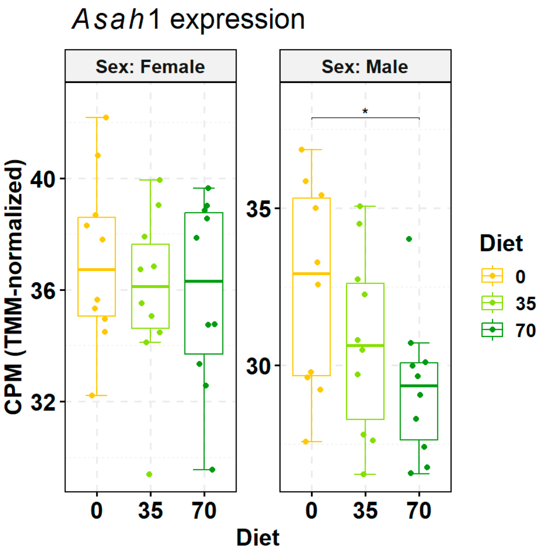

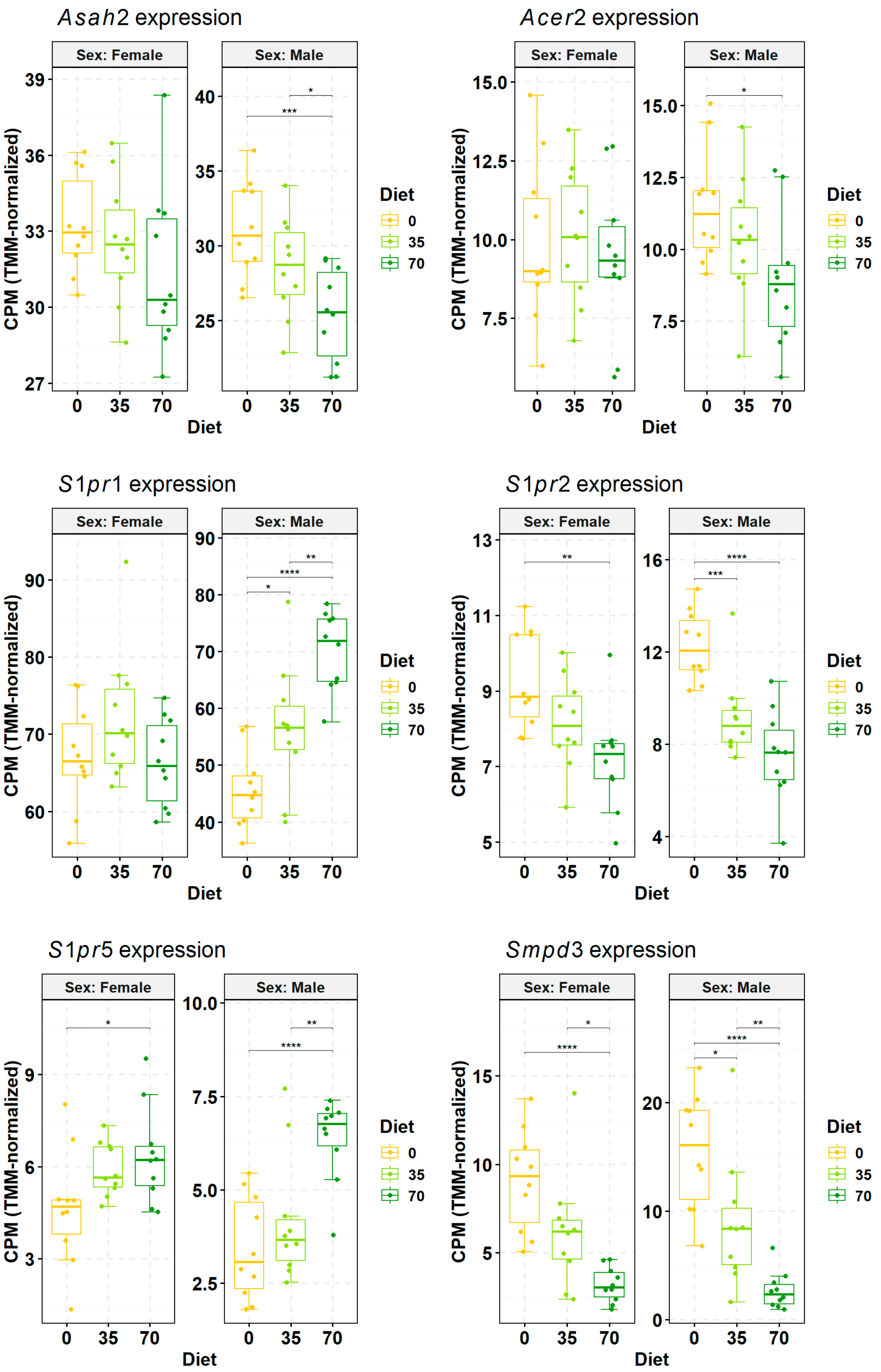

Asah2 expression
Acer2 expression
S1pr1 expression
S1pr2 expression
S1pr5 expression
Smpd3 expression
Sex: Female
Sex: Male
CPM (TMM-normalized)
Diet
0
35
70

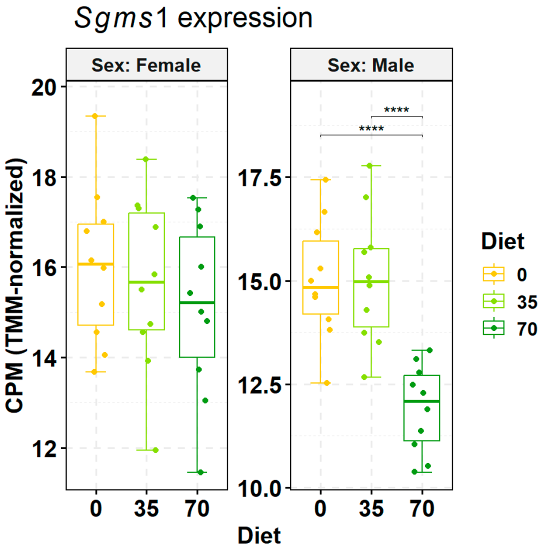

Figure S4. Box plots of genes expressed in the ceramide production from the *de novo* biosynthesis, salvage, and sphingomyelin hydrolysis pathways between females and males. Diet indicates the dose of bean expressed in the percentage of total dietary protein derived from bean. Statistically significant differences where * p-value < 0.05; ** p-value < 0.01; *** p-value < 0.001; **** p-value < 0.0001.

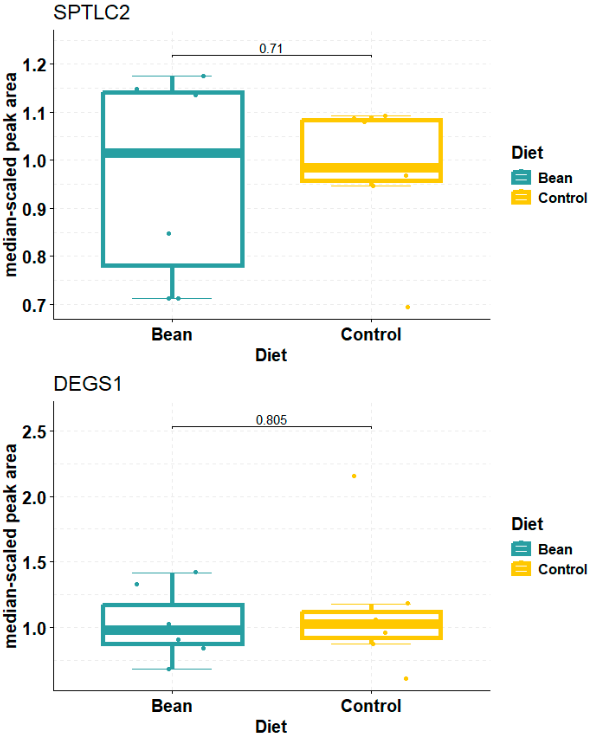

Figure S5. Box plots of protein levels in the ceramide *de novo* biosynthesis pathway in males. Statistical *p*-values are included.

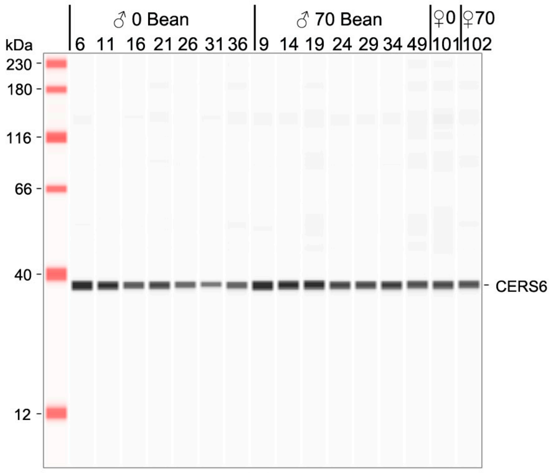

(a)

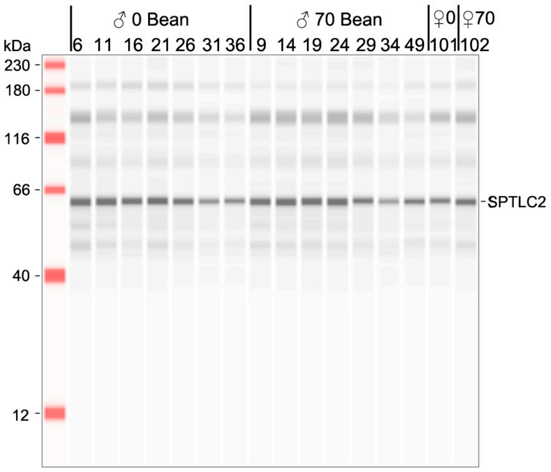

(b)

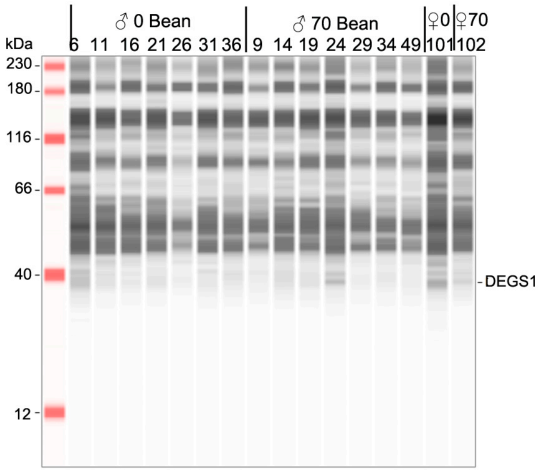

(c)

Figure S6. Western blot gel images of protein levels in the ceramide *de novo* biosynthesis pathway in the liver. Sample information on group and sex is on top of each panel. Numbers underneath indicate sample IDs. Pooled female samples are included.
